# Depression and Its Phytopharmacotherapy—A Narrative Review

**DOI:** 10.3390/ijms24054772

**Published:** 2023-03-01

**Authors:** Lukasz Dobrek, Krystyna Głowacka

**Affiliations:** Department of Clinical Pharmacology, Wroclaw Medical University, 50-556 Wroclaw, Poland

**Keywords:** depression, treatment, medicinal plants, herbal, antidepressant

## Abstract

Depression is a mental health disorder that develops as a result of complex psycho-neuro-immuno-endocrinological disturbances. This disease presents with mood disturbances, persistent sadness, loss of interest and impaired cognition, which causes distress to the patient and significantly affects the ability to function and have a satisfying family, social and professional life. Depression requires comprehensive management, including pharmacological treatment. Because pharmacotherapy of depression is a long-term process associated with the risk of numerous adverse drug effects, much attention is paid to alternative therapy methods, including phytopharmacotherapy, especially in treating mild or moderate depression. Preclinical studies and previous clinical studies confirm the antidepressant activity of active compounds in plants, such as St. John’s wort, saffron crocus, lemon balm and lavender, or less known in European ethnopharmacology, roseroot, ginkgo, Korean ginseng, borage, brahmi, mimosa tree and magnolia bark. The active compounds in these plants exert antidepressive effects in similar mechanisms to those found in synthetic antidepressants. The description of phytopharmacodynamics includes inhibiting monoamine reuptake and monoamine oxidase activity and complex, agonistic or antagonistic effects on multiple central nervous system (CNS) receptors. Moreover, it is noteworthy that the anti-inflammatory effect is also important to the antidepressant activity of the plants mentioned above in light of the hypothesis that immunological disorders of the CNS are a significant pathogenetic factor of depression. This narrative review results from a traditional, non-systematic literature review. It briefly discusses the pathophysiology, symptomatology and treatment of depression, with a particular focus on the role of phytopharmacology in its treatment. It provides the mechanisms of action revealed in experimental studies of active ingredients isolated from herbal antidepressants and presents the results of selected clinical studies confirming their antidepressant effectiveness.

## 1. Introduction

Depression is a major mood disorder presenting with a persistent feeling of sadness, debilitating low mood, impaired cognition and loss of interest. Depression has a profound effect on the functioning of the affected person, individually, biologically and socially. Depression involves deep sadness, hopelessness, sorrow, emptiness and despair. Over time, it may also involve an inability to experience pleasure, psychomotor dysfunction, changes in sleep and eating behaviours, difficulty concentrating and suicidal thoughts [1]. In fact, depression belongs to a heterogeneous group of diseases, broadly included in the International Classification of Diseases (ICD) published by the World Health Organization (WHO). The current ICD-11 version distinguishes a few depressive disorders: single episode depressive disorder (moderate, without psychotic symptoms, or severe, with or without such symptoms) and recurrent depressive disorder (current episode moderate, without psychotic symptoms, or severe, with or without such symptoms, or recurrent depressive disorder currently in full remission, or unspecified recurrent depressive disorder). Moreover, there are other different forms of depression, such as dysthymic disorder (persistent depressive disorder), mixed depressive and anxiety disorder, other specified depressive disorders and unspecified depression [2].

As presented in this review, depression is currently one of the most important diseases of civilization and a significant public health problem. Therefore, it seems important to perform a periodic, comprehensive analysis focusing on the description of this disease and its therapeutic management options, taking into account phytopharmacotherapy, which is less popular in everyday clinical practice.

This paper aims to briefly summarize the most important issues concerning the epidemiology, pathophysiology, symptomatology and treatment of depression. It also discusses the importance of phytopharmacotherapy in treating this disease and provides an outline of the phytopharmacodynamics of medicinal plants with antidepressant activity, with particular emphasis on the importance of their anti-inflammatory effect. This narrative review employs the traditional, non-systematic literature review method (PubMed, Google Scholar databases) with the use of the following search terms and their combinations: “depression”, “epidemiology”, “pathophysiology”, “symptomatology”, “management”, “treatment”, “medicinal plants”, “phytopharmacotherapy”, “phytopharmacodynamics”. The selection of relevant articles for review based on their titles and abstracts by one author (LD) was supervised critically by the second author (KG). Both review articles and original full-text articles were taken into account, preferring search results from the last ten years, but also including older papers, which, according to the authors, introduced important information to the discussion. At the same time, the performed literature screening revealed some papers analogous to our forthcoming review, e.g., Pardhe et al. [3] or Martins and Brijensh [4] and others. These papers describe the phytopharmacodynamics of many different plants with antidepressant activity, mostly focusing on their effect on disturbances of neurotransmission in the CNS found in depression. However, we made efforts to prepare a comprehensive review, discussing phytopharmacotherapy of depression against the background of a broader introduction to the epidemiology, symptomatology and pathophysiology of this disease, focusing the description of phytopharmacodynamics also on other aspects less frequently addressed in other papers, such as the contribution of the anti-inflammatory properties to the antidepressant effect.

## 2. Epidemiology

According to the WHO (data as of 13 September 2021), depression affects 3.8% of the world’s population, including 5.0% of adults and 5.7% of adults over 60 years. Approximately 280 million people worldwide suffer from depression. The disease is a leading cause of disability worldwide and is a major contributor to the overall global burden of disease. More women are affected by depression than men [5]. A population-based study in Europe using data from 27 countries collected between 2013 and 2015 showed that the overall prevalence of the current depressive disorder is high (6.38%), with important variation across European countries, ranging from 2.58% in the Czech Republic to 10.33% in Iceland. Similarly to the WHO data, the study demonstrated higher depression prevalence in women (7.74%) compared to men (4.89%), with clear gender differences for all countries, except Finland and Croatia [6]. Estimates for Poland indicate that around 1.5 million people suffer from depression, and this disease affects approximately 3% of people of productive age (i.e., 766,000 adult Poles had at least one depressive episode in their lives) [7].

Outside Europe, the prevalence of depression is equally high. According to the National Center for Health Statistics of the Centers for Disease Control and Prevention (CDC), 8.1% of adults aged 18 and over had symptoms of anxiety disorder, 6.5% of depressive disorder and 10.8% of anxiety disorder or depressive disorder in the USA in 2019 [8]. In the Asia-Pacific region, the prevalence of 1-month major depression ranged from 1.3% to 5.5%, and rates of major depression ranged from 1.7% to 6.7% [9]. Ogbo et al. estimated the prevalence of depressive disorders in South Asia as high as 3.9%, 4.4% in Bangladesh, 3.9% in India, 3.0% in Pakistan, 4.0% in Nepal and 3.7% in Bhutan [10]. In Latin America and the Caribbean, depression affects 5% of the adult population. Moreover, six out of every ten people do not receive treatment [11]. In South Africa, an estimated 9.8% of the adult population experience major (clinical) depression at some point in their life [12]. The pooled prevalence of depression among older adults in Africa was estimated to be even higher, reported at 26.3% [13]. In Australia, 9.4% of males aged 16–85 and 12.8% of females of the same age experienced a depressive episode in their lives [14].

Thus, it should be concluded that depression is a serious disease of civilization. As early as 2006, the WHO estimated that depression would cause the second largest increase in morbidity after cardiovascular diseases and pose a significant public health challenge [15].

## 3. Pathophysiology

There are some biological (including genetic abnormalities, microbiome disturbances, inflammatory factors, stress and dysfunction of the hypothalamic–pituitary–adrenal (HPA) axis and the kynurenine pathway), psychological and social determinants of depression. Depression may also secondarily develop in the course of many somatic or mental diseases. In fact, depression, or collectively named depressive disorders, cannot be explained by a single theory since many variables are involved in the entity’s initiation and sustainment. This paper does not provide a detailed description of the pathophysiology of depression, which can be found in numerous reviews on this issue. Only selected aspects of the pathophysiology of depression are briefly mentioned below.

There are some biological theoretical frameworks for the explanation of the onset of depression. The most common biochemical, neurophysiological explanation for depression is the deficit of monoamines (serotonin, noradrenaline, dopamine), which play a key role in important life-regulating functions (appetite, sleep, memory, learning, temperature regulation, social behaviour). The insufficiency of these monoamine neuromodulators in definite structures of the central nervous system is considered to be responsible for the development of depression [16]. This monoamine hypothesis of depression was historically the first theory proposed by Joseph Schildkraut in the 1960s and was based on the successful use of iproniazid (a monoamine oxidase inhibitor) and imipramine (a monoamine neuromodulator reuptake inhibitor) in the treatment of depression [17,18,19]. This theory is consistent with clinical observations—designed tricyclic antidepressants and monoamine neuromodulator reuptake inhibitors have confirmed the important role of imbalance and neuromodulator deficiency. For many years, the monoamine theory was the basic paradigm setting the ground rules in the treatment of depression.

Moreover, the stress-induced overactivity of the HPA axis was also revealed to be involved in the pathophysiology of depression. Significant correlations between measures of stress and depressive behaviour and between cortisol levels and depressive behaviour were found in experimental studies [20]. Influencing the HPA axis and reducing its activity may become another therapeutic option in treating depression [21]. This direction seems to be particularly interesting and promising, especially considering the secondary relationship between the activity of the HPA axis and the gut microbiota. It is believed that the gut microbiota can influence the HPA axis function through the activity of cytokines, prostaglandins or bacterial antigens of various microbial species [22].

Also, both experimental and clinical studies indicate that inflammatory processes may play a causal role in the development of depressive illness.

There is growing evidence that immune system disturbances are involved in the development of depression. Various immune cytokines released during systemic, “low grade” and self-sustaining inflammation have been found to be implicated in the pathophysiology of depression, including interleukins (IL)-1, IL-2, IL-4, IL-6, IL-8 and IL-10; interferon-gamma (IFN-γ); C-reactive protein (CRP); tumour necrosis factor-alpha (TNF-α); and monocyte chemoattractant protein-1 (MCP-1) [23,24].

The peripherally released cytokines may pass the blood–brain barrier, activating glial cells and leading to a neuroinflammatory process contributing to brain damage [24,25]. There is also evidence that central neurotransmission disturbances are associated with secondary disturbances concerning relevant cytokines, e.g., serotonin deficiency, contributing to the feeling of sadness, guilt and worthlessness and disturbed appetite related to Il-6, Il-18, TNF-α and CRP abnormalities. Sociability dysfunction, due to lower dopamine levels, was demonstrated to correlate with the disturbances within INF-γ, Il-17, Il-33 and CCR6 and impaired functioning of Th1 and Th17 cells. Some symptoms depend on multiple neurotransmitters, such as psychomotor retardation (manifested by INF-γ, TNF-α, Il-1β and Il-6 disturbances) regulated by serotonin, dopamine, norepinephrine and glutamate [26]. It is noteworthy that antidepressant treatment affects the level of cytokines. A meta-analysis of 32 clinical studies by Więdłocha et al. [27] demonstrated significant decreases in IL-4, IL-6 and IL-10 in major depressive disorder (MDD) subjects after antidepressant treatment. In the case of IL-1ß, the decrease was significant exclusively for SSRI drugs. Moreover, the activation of the kynurenine pathway and reduced tryptophan levels correlate with inflammation-induced depression, as the kynurenine pathway is believed to precipitate depressive symptoms by depleting brain serotonin [28]. Multiple endogenous and environmental factors appear to increase the risk of developing depression and seem to be associated with systemic inflammation; these include psychosocial stressors, poor diet, physical inactivity, obesity, smoking, altered gut permeability, atopy, dental caries, sleep and vitamin D deficiency [29]. Oxidative stress (OS) is a supplementary mechanism involved in the pathophysiology of depression because OS is closely related to the inflammatory process. In the course of an inflammatory process, positive reciprocal action is established—inflammatory mediators intensify the synthesis of free radicals (mainly reactive oxygen/nitrogen species), which in turn sustains inflammation and the release of pro-inflammatory mediators. The limbic brain regions (prefrontal cortex, hippocampus and amygdala) involved in mood and behaviour control are highly susceptible to oxidative damage. Previous studies mention the implication of OS in neurodegenerative and psychiatric disorders, including depression [30,31]. Moreover, excessive and prolonged stress negatively impacts the immune system, which in turn affects the HPA axis. Both factors lead to neurological impairments in the brain, causing changes in mood and behaviour [32].

There is also a relationship between systemic inflammatory alterations and gut microbiota. The gut and brain are two structures connected at multiple levels. The microorganisms inhabiting the gut and their products are essential in this bidirectional communication, conforming to the microbiota–gut–brain (MGB) axis [33,34]. Depressed patients show significant changes to the gut microbiota (dysbiosis) in comparison to healthy patients, leading to a pro-inflammatory status and neuroinflammation, enhancing the HPA axis dysfunction and stress sensitivity in the brain and disrupting the gut–brain communication through the vagus nerve, hence contributing to the pathogenesis of MDD [35]. In addition, an altered immune status described in MDD is responsible for an enhanced bacterial translocation in the bloodstream, aggravating the systemic damage in depressed patients [36]. Moreover, there is growing evidence of an important role of gut microbiota in the production or degradation of multiple neurotransmitters, including serotonin, norepinephrine, dopamine or gamma-aminobutyric acid (GABA) [37], defining the gut microbiota as a critical modulator of brain activity.

The contribution of reproductive hormones to mood has also been a focus of efforts to explain the detailed pathophysiology of depression. Recent longitudinal studies have found that women are more susceptible to higher levels of depressed mood during the menopausal transition than just before it starts, suggesting differences in the prevalence of depression in relation to the sex of the patients [38]. In addition, significant decreases in oestrogen production, an overall state of hypogonadism, stability in the hypothalamic–pituitary–gonadal axis and elevated FSH are marks of menopause. The decreased circulating androgen levels associated with menopause have also been linked to the loss of libido, fatigue and an increase in depressive symptoms [39].

Family and twin studies have provided strong evidence for the involvement of genetic factors in the risk of depression. Twin studies have demonstrated that the heritability rate of depression is about 37%, and data from family studies indicate a two- to three-fold increase in the risk of depression in the first-degree offspring of depressed patients [40]. Heredity has also been shown to particularly affect severe forms of depression [41].

In most cases of depression, estimates indicate that about 50% of the causes are genetic, and about 50% are unrelated to genes (psychological or physical factors). Genetic background is especially suspected in patients whose parent or sibling has suffered from depression more than once (“recurrent depression”) and if the depression started relatively early in life (in childhood, teenage years or twenties). However, there is no one “depressive gene”. Some of the possible genetic causes include the role of polymorphisms in genes related to the neurotransmission of serotonin, norepinephrine and dopamine, such as serotonin transporter gene variants that inhibit serotonin reuptake, leading to a deficiency of monoamines in the brain and thus predisposing to depression. Another possibility is a polymorphism in genes regulating nervous system development, leading to a deficiency in the number of neurons in the adult brain or in genes regulating anti-inflammatory cytokines secreted in a compensatory manner to counteract inflammation. Further, genes that regulate circadian rhythms are another potential cause of genetic predisposition to depression by interfering with normal sleep and other bodily functions that depend on the circadian pacemaker.

Furthermore, in terms of genetic abnormalities, there are also links between genetic factors and depression; for example, abnormalities in brain-derived neurotrophic factor (BDNF) appear to play an important role in depression. The “BDNF theory” of depression results from preclinical studies demonstrating that several forms of stress reduce BDNF-mediated signalling in the hippocampus, whereas chronic treatment with antidepressants increases BDNF-mediated signalling. Treatment with antidepressants increases several growth factors in the hippocampus that influence neurogenesis. These include BDNF (which promotes neuronal survival) and vascular endothelial growth factor (VEGF) [42]. However, there are also studies revealing that male mice with conditional forebrain deletions of BDNF or its receptor do not show depression-like behaviour [43]. Moreover, the action of BDNF may be brain region dependent—in the ventral tegmental area (VTA) and nucleus accumbens (NAc), BDNF exerts a potent pro-depressant effect, and the direct infusion of BDNF into the VTA–NAc increases depression-related behaviours [44]. These results suggest that the current formulation of the BDNF hypothesis of depression development is too simplistic. BDNF-mediated signalling is involved in neuroplastic responses to stress and antidepressants, but these effects are both region- and antidepressant-specific [42]. In the neurobiology of depression, at the cellular and molecular levels, a number of signalling pathways and targets have been suggested as implicated in the pathogenesis of depression, including the above-mentioned neurotrophic factor and glycogen synthase kinase 3 (GSK3) pathways. The functional consequences of these systems in the context of the damaging effects of chronic stress, including atrophy and loss of neurons and glia, were also observed in brain imaging and postmortem studies of depressed patients [45].

In addition, there are links between genes of the core region of the tissue compatibility system, as well as various gene polymorphisms and depression. Single nucleotide polymorphisms (SNPs) of genes involved in the tryptophan catabolism pathway are also being investigated [46,47,48].

An important role in the current description of depression is also played by epigenetics, i.e., the science dealing with inherited changes in gene expression unrelated to changes in the DNA sequence, examining the mechanisms of interaction between genes and their products in phenotype formation. Thus, epigenetics is the study explaining the cellular control of gene activity without changing the DNA sequence [49]. Epigenetic mechanisms include histone acetylation, which changes the structure of chromatin; cytosine methylation in DNA (in areas rich in the sequence of dinucleotides (cytosine-phosphate group-guanine)), which prevents gene transcription; and the influence of the non-coding microRNA binding complementary to mRNA, thus regulating translation [50]. Experimental studies indicate that genetic and environmental risk factors and their interactions induce aberrant epigenetic mechanisms targeting stress response pathways, neuronal plasticity and other behaviourally relevant pathways involved in major depression. The role of epigenetics in depression pathogenesis would explain the differences in the incidence of this disease in monozygotic twins. The involvement of epigenetic mechanisms in depression pathogenesis also offers an explanation of largely inconsistent genetic association studies of depression, for example, by undermining the transcriptional impact of DNA sequence polymorphisms due to epigenetic modifications on those gene promoters [42,51].

In addition, growing clinical data indicate that the analysis of epigenetic changes in patients with depressive disorders can be not only a marker of clinical improvement, but also a predictor of response to pharmacological treatment. It is suggested that the use of histone deacetylase inhibitors (natural or synthetic small molecules that can inhibit the activity of deacetylases and affect the availability of chromatin for transcription factors) may become a novel method of treating depression and other affective disorders [52].

Finally, the description of the pathophysiology of depression also takes into account social and psychological issues. According to attachment theory, depression is determined by a person’s inability to establish strong and long-lasting affective bonds with other people. The attachment model postulates that vulnerability to depression stems from early experiences that did not meet the child’s need for security, care and comfort, as well as the current state of their intimate relationships. The links between secure attachment and depression also appear to be mediated by the development of maladaptive beliefs or schemas [16]. Complex and incompletely understood psychological and social maladjustment can result in anaclitic depression, which arises from feelings of loneliness and abandonment, and introjective depression, which stems from a sense of failure and worthlessness [53].

There are also studies focusing on the importance of circadian rhythms and its main mediator, melatonin, in the onset and development of the disease [54]. Based on this hypothesis, some melatonin receptor agonists (ramelteon, tasimelteon) have been introduced into clinical practice in the treatment of sleep disturbances, and those acting additionally as serotonergic antagonists (agomelatine), which display antidepressant properties [55].

**Figure 1:**
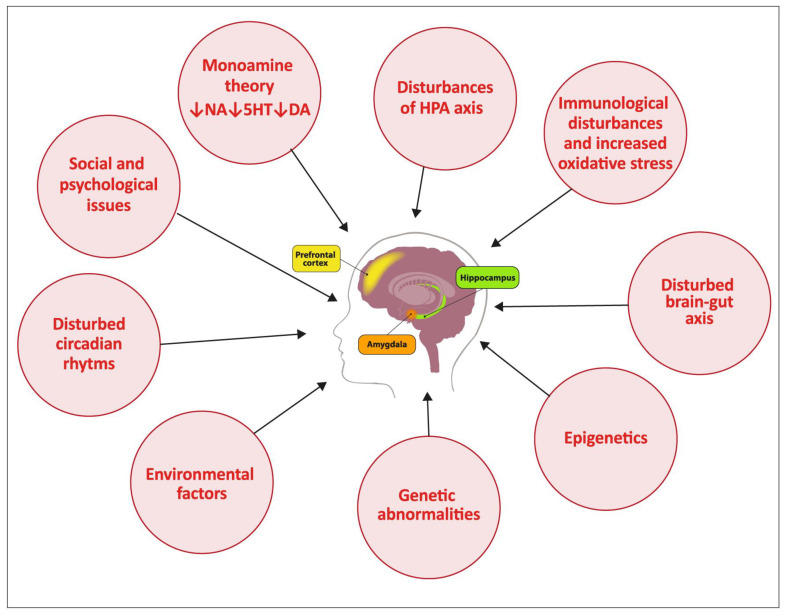
The main pathomechanisms of depression. The main structures associated with the pathogenesis of depression are the prefrontal cortex and the limbic system (including the hippocampus and the amygdala). According to the comprehensive, psycho-neuro-immuno-endocrinological theory of the development of depression, this disease results from the action of multiple exogenous and endogenous factors. Details are given in the text. (HPA—hypothalamic-pituitary-adrenal axis; 5HT—5-hydroxytryptamine; serotonin; NA—noradrenaline; DA—dopamine).

To sum up, the pathophysiology of depression is multifactorial, and the treatment of this mental illness remains a challenge. Many causative, interrelated factors are implicated in depression pathogenesis, as shown in Figure 1.

To underline its complexity, a “psycho-neuro-immuno-endocrinological” term has been introduced to describe depression [56]. The main pathomechanisms of depression have focused on impaired monoamine function, decreased monoamine production, malfunction of the secondary messenger system or changes in other neurotransmissions. A significant role in the pathogenesis of depression is also attributed to inflammation and oxidative stress, which exert a major influence, affecting the proper functioning of the brain. Additional attention has also been given to endocrine abnormalities (excessive cortisol levels) or impaired neurogenesis through reduced levels of the brain-derived neurotrophic factor. The role of abnormal circadian rhythm is also highlighted [15].

## 4. Symptomatology

Depression manifests itself in a variety of both somatic and psychological symptoms. The disease also has a huge impact on the social and professional functioning of the patient. The typical psychological symptoms of depression include continuous low mood or sadness, with a dominant feeling of hopelessness, helplessness and guilt, feeling worried and/or anxious, poor concentration, lack of motivation to undertake everyday activities and loss of previous interests. In addition, patients with depression may feel overwhelmed, restless or angry and lack confidence. The somatic symptoms include sleep abnormalities, such as insomnia or hypersomnia (many patients experience early morning awakenings; there are also patients who tend to feel sleepy during the day), changes in appetite or body weight (usually reduced, but sometimes increased), feelings of low energy or adynamia, low sex drive (loss of libido), changes in the menstrual cycle and constipation. Social symptoms of depression include avoiding contact with friends and participating in fewer social activities; neglecting hobbies and interests; and difficulties at home, work or family life as a result of chronic emotional disorders affecting the ability to maintain family contacts and professional activity. Usually, depressive patients may also present altered behaviour, such as staying in rather than going out and being less productive at school or work. Depression may also take on an atypical form, manifesting itself by increased mood reactivity (i.e., mood brightens in response to positive events) and increased appetite; sleeping longer; leaden paralysis (i.e., heavy, leaden feelings in arms or legs); and interpersonal rejection sensitivity (not limited to episodes of mood disturbance), resulting in significant social or occupational impairment [57]. Depending on the number and severity of the above-mentioned symptoms, depression can be mild, moderate or severe, with possible suicide attempts. As an aside, it should also be mentioned that depression in some patients may be “masked”, especially in the form of purely somatic disorders in the elderly [58,59,60]. Noteworthy, increased alcohol dependence was demonstrated in the course of depression—the prevalence of depression among alcohol-dependent persons is high (estimated at 63.8%) [61].

## 5. Treatment—Pharmacotherapy and Non-Pharmacological Management of Depression

The treatment of depression involves both pharmacological and non-pharmacological methods, including, in particular, techniques of therapeutic psychotherapeutic influence. The concept of “collaborative care” is the basis for the comprehensive treatment of depression. Psychological therapy should be the main treatment for mild depression or complementary to pharmacological treatment in other cases. There is strong evidence for the effectiveness of combined pharmacological antidepressants and cognitive behaviour therapy over the sole use of antidepressants in moderate to severe depression and chronic depression [62].

There are several classes of antidepressants used in the pharmacotherapy of this disorder, including selective serotonin reuptake inhibitors (SSRIs), tricyclic antidepressants (TCAs), serotonin and noradrenaline reuptake inhibitors (SNRIs), noradrenaline reuptake inhibitors (NRIs) and noradrenaline and dopamine reuptake inhibitors (NDRIs). These drugs inhibit the transporters responsible for the reuptake of monoamines [63]. There are also other drugs with antidepressant effects (e.g., agomelatine, an MT1 I MT2 melatonin receptor agonist and serotonin 5HT2 receptor antagonist, or mirtazapine, an antagonist of adrenergic alpha2-autoreceptors, alpha2-heteroreceptors, 5-HT2 and 5-HT3 receptors) [63]. Recent antidepressants include desvenlafaxine, levomilnacipran, vortioxetine or vilazodone [64]. Selective serotonin reuptake inhibitors (SSRIs) are considered by general practitioners to be first-line drugs in the treatment of outpatients with depression. All antidepressants are regarded to be more effective than a placebo in adults treated for depression. In a systematic review and meta-analysis by Cipriani et al. [65], agomelatine, amitriptyline, escitalopram, mirtazapine, paroxetine, venlafaxine and vortioxetine were found to be more effective than other antidepressants, while fluoxetine, fluvoxamine, reboxetine and trazodone were found to be the least effective. However, larger differences in the efficacy and acceptability of individual antidepressants were revealed in head-to-head trials. For acceptability, agomelatine, citalopram, escitalopram, fluoxetine, sertraline and vortioxetine were better tolerated than other antidepressants, while amitriptyline, clomipramine, duloxetine, fluvoxamine, reboxetine, trazodone and venlafaxine had the highest dropout rates [65].

Taking into account the clinical picture of depression, the response to initial treatment and the patient’s comorbidities, a precise choice of medication is made, and the effect of treatment is assessed after an appropriate period of follow-up. It should be stressed that antidepressants, like other pharmacological agents, exert some adverse drug reactions (ADRs). The most common ADRs observed in patients treated with SSRIs (e.g., paroxetine, sertraline, fluoxetine, escitalopram) at the primary care outpatient clinics were: gastrointestinal problems (in 17% of subjects), indigestion (22%), nausea (18%), diarrhoea (9%) and constipation (11%). Moreover, tiredness (in 45% of subjects), dizziness (24%), hypotension (15%), headache (34%) and blurred vision (22%) were also reported [66]. SSRIs are generally better tolerated than other antidepressants. The less common ADRs reported in the literature include extrapyramidal symptoms (EPS), serotonin syndrome, QT prolongation, rash, birth defects, hyponatraemia and cataracts [67]. Tricyclic antidepressants show more pronounced side effects due to their complex mechanism of action and receptor non-selectivity. The most common adverse effects include constipation, dizziness and xerostomia. Due to their cholinolytic potential, TCAs may also produce blurred vision, constipation, xerostomia, confusion, urinary retention and tachycardia. Moreover, due to the blockade of alpha-1 adrenergic receptors, orthostatic hypotension and dizziness may develop. TCA-induced histamine blockade (H1) contributes to sedation, increased appetite, weight gain and confusion. TCAs may also cause cardiovascular complications, including arrhythmias, such as QT prolongation, ventricular fibrillation and sudden cardiac death in patients with pre-existing ischaemic heart disease. In addition, treatment with TCAs may be associated with mild liver enzyme elevation [68].

Detailed recommendations for pharmacotherapy of depression are beyond the scope of this paper and can be found in numerous guidelines, including those published by psychiatric scientific societies [69,70,71,72], such as the Polish Society of Psychiatry [73].

Non-pharmacological interventions also play an important complementary role in the comprehensive treatment of depression. They include primarily psychotherapeutic techniques (e.g., cognitive behavioural therapy, naturopathic therapy, physical activity interventions or acupuncture) [74,75]. Some studies demonstrate the benefits of some dietary supplements on depressed mood. They are based on the polyunsaturated fatty acids (PUFAs), combining eicosapentaenoic acid (EPA) and docosahexaenoic acid (DHA), and probiotics, which is in line with the assumption that inflammation and dysfunction of the gut–brain axis are pathogenetic elements of depression [76]. Other highly promising dietary interventions studied for potential use in depressive patients involve a specific group of nutrients (vitamins, polyphenols and caffeine), foods (fish, nuts, fruit seeds and vegetables, coffee/tea and fermented products) or dietary supplements (such as S-adenosylmethionine, acetylcarnitine, creatine, amino acids, etc.) [77]. In severe cases of depression refractory to classical pharmacological treatment, advanced non-pharmacological techniques, such as repetitive transcranial magnetic stimulation (rTMS) or electroconvulsive therapy (ECT), are also used [78,79]. Systematic reviews and meta-analyses demonstrated that both techniques are effective in depression treatment, with ECT superior compared to rTMS. Although ECT was the most efficacious, it was the least tolerated treatment, while rTMS was the best-tolerated treatment for MDD [80,81].

## 6. Depression Phytopharmacotherapy as an Alternative to Classical Antidepressant Treatment/Examples of Preparations and General Reasons for Their Antidepressant Effect

There is now an upward trend in the number of prescriptions for antidepressants globally. The number of prescriptions for antidepressants in England has almost doubled over the past decade. As many as 70.9 million prescriptions for antidepressants were registered in 2018, up from 36 million in 2008 [82]. From 2009–2010 through 2017–2018, the proportion of adults treated with antidepressants also increased in the USA. According to an analysis by the National Center for Health Statistics, in 2017, the percentage of the US population over the age of 12 years who had taken antidepressants in the past month was estimated at 12.7% [83]. According to “The state of mental health in America 2022” [84], 15.08% of youth experienced a major depressive episode in the past year, and 24.7% of adults with a mental illness reported an unmet need for treatment. European data also indicate high use of antidepressants. In a large general population study from 27 European countries that measured antidepressant use and regularity of use, 7.2% of participants reported taking antidepressants in the past year. There were large differences in the prevalence of antidepressant use between countries, ranging from 15.7% in Portugal to 2.7% in Greece. The top five European countries in terms of the use of antidepressants in the last 12 months were Portugal, Lithuania, Malta, the UK and France. In contrast, the five countries with the lowest use of antidepressants in the last 12 months were Greece, Germany, Bulgaria, Cyprus and the Czech Republic. In this respect, Poland was ranked 19th among the 27 countries assessed. In contrast, the countries with the highest proportion of patients regularly taking antidepressants were Sweden, the United Kingdom, Denmark, Finland and the Netherlands. The countries with the lowest percentage of patients regularly using antidepressants were Bulgaria, Romania, the Czech Republic, Lithuania and Slovakia. In this respect, Poland also took 19th place in the ranking (out of all 27 countries assessed) [85]. It should be strongly emphasized that antidepressants are drugs that produce significant, numerous adverse drug reactions, especially in patients using polypharmacy. The most common adverse effect reported by patients was weight gain after TCAs, followed by sexual dysfunction for SSRIs, nausea or vomiting for monoamine oxidase inhibitors (MAOIs) and headache for SNRIs [86]. Notably, TCAs were associated with a wide range of ADRs, such as toxic delirium, grand mal seizures, increased liver enzymes, urinary retention, flushing or cardiovascular disorders (i.e., mainly orthostatic collapse). Psychological and neurological ADRs were the most common in SSRI-treated patients, followed by gastrointestinal, dermatological and endocrine/electrolyte reactions, with agitation, hyponatraemia, increased liver enzymes, nausea and serotonin syndrome as leading adverse effects [87]. In the study by Uher et al. [88], ADRs induced by nortriptyline or escitalopram were assessed on the basis of the Antidepressant Side-Effect Checklist and the psychiatrist-rated UKU Side Effect Rating Scale. Dry mouth (74%), constipation (33%) and weight gain (15%) were associated with nortriptyline treatment. Diarrhoea (9%), insomnia (36%) and yawning (16%) were more common during treatment with escitalopram. Problems with urination and drowsiness predicted discontinuation of nortriptyline, while diarrhoea and decreased appetite were the main causes of discontinuation of escitalopram.

Given the high use of antidepressants and their possible side effects, other treatment options for depression are being explored, including the use of herbal medicines. Therefore, phytopharmacotherapy is a promising therapeutic option that appears to be a safer alternative, particularly for patients with mild depressive disorders or for seasonal dysthymia (“winter depression”).

For centuries, people have tried to treat depression with available remedies of natural origin used as part of traditional medicine. In different cultures and geographic regions, certain medicinal plants have been known and used to treat many different conditions. Estimates indicate that of the more than 300,000 seed plants, approximately 60% have been used for their medicinal properties [89]. In some regions (especially Africa, South America and Asia), the use of traditional medicine systems (including medicinal plants) based on social and ethnic continuity and empirical findings is the main therapeutic approach. Ethnomedicine (ethnopharmacology) has also distinguished medicinal plants as effective in the treatment of neurological and psychiatric disorders [90]. In summary, medicinal plants (“herbs”) contain various pharmacologically active compounds in their tissues: alkaloids, glucosides, essential oils, fatty oils, mucilages, tannins, gums, flavonoids, iridoids and bitters, saponins and others that cannot be separated into individual compounds. This fact distinguishes the mode of action of phytopharmaceuticals from classical, synthetic drugs—the pharmacological action mediated by phytopharmaceuticals is not mediated by just one compound, but is the result of the synergistic and polyvalent, complementary action of many active substances. On the contrary, the “mainstream” pharmacodynamic effect in classical pharmacology is based on an isolated, single active compound. A synergistic effect is defined as an effect produced by a combination of substances that is greater than would be expected if the combined action of the individual components were considered [15,91,92]. A complementary concept is the theory of the polyvalent action of phytopharmacological ingredients, which assumes that herbal extracts can exert a wide range of biological activity due to the variety of chemical compounds present in herbs, each of which produces different effects [15,93].

The synergistic and polyvalent effects of herbal compounds in the treatment of depression and other mental disturbances are becoming increasingly important. In a study by Kessler et al. [94], 54% of patients suffering from depression reported using herbal medicines in the past 12 months to treat their disorder. Similar to this finding, it was revealed that 44% of psychiatric inpatients hospitalized for acute care for various psychiatric disturbances had used herbal medicines in the previous 12 months [95]. Despite the popularity of herbal medicines in the treatment of depression, as well as other psychiatric disorders (such as anxiety or insomnia), research on phytopharmaceuticals in neuropsychology is not as advanced as for synthetic drugs. For the most part, the results of beneficial effects of phytopharmaceuticals in the treatment of nervous system disorders have been obtained in vitro or in preclinical studies in laboratory animals, with an abundance of clinical studies validating the efficacy and safety of phytopharmaceuticals in patients [15,96]. There is also well-established use of herbal medicines containing active substances dating back more than ten years, and their efficacy and safety have been well-established, so the use of such preparations is legally possible based on the results obtained from a review of the scientific literature. In addition, there is also traditional use of herbal medicines containing plants or parts or extracts of plants that have been traditionally used for centuries, and their administration for various clinical conditions is based on empirical evidence, which means that they are acceptably safe, although they do not have a precisely defined level of efficacy [97,98].

Most herbal medicines used in the phytopharmacology of depression are over-the-counter (OTC) preparations or dietary supplements and are considered safe and induce fewer ADRs compared to conventional medicines, especially TCAs (cholinolytic symptoms, sexual dysfunction, insomnia, withdrawal problems) [15,99,100,101]. Examples of OTC drugs or dietary supplements used for depression in Poland (the country of residence of the authors of this review) are summarized in Table 1. The examples of preparations listed there indicate that the most popular antidepressant preparations in Poland are based on St. John’s wort and saffron crocus, with the possible addition of lemon balm, B vitamins or amino acids that are sources of monoamines (tryptophan, phenylalanine).

Preparations with similar compositions are used in other countries. In general, commercially available OTC drugs or dietary supplements usually contain various nutraceuticals, such as vitamins (including vitamin D and vitamin B group); S-adenosyl methionine (considered the universal methyl donor in living organisms); amino acids (phenylalanine, tyrosine and tryptophan); amino acids that are precursors of neurotransmitters (noradrenaline, serotonin); microelements (zinc, magnesium); and phytoceuticals (St. John’s wort, saffron crocus, turmeric, roseroot, lavender), often with the addition of adaptogenic ashwagandha and anxiolytic kava [102].

Unlike preparations in the Polish pharmaceutical market, foreign products are often enriched with omega-3 acids (e.g., EPA and DHA) because these nutrients can reduce inflammation in the brain, which may positively impact mood. Moreover, turmeric is rarely found in commercially available preparations popular in the Polish pharmaceutical market. Moreover, there is no preparation containing *Piper methysticum* (kava) in Poland due to the warnings issued by the European Safety Food Authority (ESFA) about the potential hepatotoxicity of kavalactones. Thus, kava cannot be a component of dietary supplements or OTC drugs in Poland, and its use in pharmaceuticals has been prohibited. However, later studies showed the hepatotoxicity of kava preparations obtained by extracting whole plants with organic solvents, while daily intake of kavalactones in the form of tablets obtained from a traditional aqueous plant extract was not harmful [103]. This resulted in the lifting of restrictive regulations on the import and trade of kava-based pharmaceutical products. Nowadays, kava trade is regulated by each country individually.

In the further part of our review, we discuss the phytopharmacodynamics of the following plants with antidepressant activity: St. John’s wort (SJW), saffron crocus, lemon balm, lavender, gingko, Korean ginseng, roseroot, magnolia bark, borage, brahmi and mimosa tree.

## 7. Side Effects of Herbal Antidepressants Discussed in This Review

The use of herbal antidepressant preparations is characterized by greater safety compared to classic antidepressants, and this issue is one of the main advantages of phytopharmacotherapy. These preparations, which mostly have the legal status of dietary supplements, are available without a prescription and are perceived to be safe. However, all medicinal agents, including herbal preparations, have potential side effects. As with other drugs, the risk of adverse drug reactions may be influenced by a user’s age, gender, genetics, nutrition status and concurrent disease states and treatments. In clinical practice, recognizing adverse effects of herbal medicine is not routine, and their reporting is less frequent compared to synthetic drugs [104].

Among herbal antidepressants, the most recognized side effects are described for St. John’s wort, perhaps due to the fact that St. John’s wort (SJW) preparations, next to saffron-containing medicines, are the most popular plant antidepressants. The most commonly reported adverse reactions for SJW are gastrointestinal symptoms, allergic reactions, dizziness/confusion, tiredness/sedation and dry mouth. Hyperesthesia and a syndrome of dyspnoea and hyperventilation with flushing headache, mydriasis, nausea, palpitations and tremor have been also reported. The majority of these reactions were generally considered to be mild, moderate or transient. [105,106,107]. Data from observational studies have indicated that adverse events may occur in 1%–3% of patients treated with SJW preparations [108]. In the case of SJW, there is also the possibility of triggering a manic phase in the course of bipolar disorder [109]. In addition, the phytopharmacologically active components of SJW (hypericin and hyperforin) are known inducers of cytochrome enzymes (CYP1A2, 2C9, 2C19, 2D6 and 3A4, 3A2, 3E1), as well as p-glycoprotein. Therefore, chronic use of St. John’s wort is associated with a risk of pharmacokinetic interactions at the biotransformation stage with drugs whose metabolism also occurs in the cytochrome isoenzymes mentioned [110,111,112,113]. Moreover, the most widely known, possibly serious adverse effect associated with SJW administration is a fatal increase in serotonin, which can possibly cause serotonin syndrome when coupled with certain antidepressants (SSRI) and monoamine oxidase (MAO) inhibitors. It is an example of a possible pharmacodynamic SJW interaction. Serotonin syndrome is known to manifest with hyperthermia, tachycardia hypertension, mydriasis and diaphoresis [105,106]. A detailed list of possible clinically significant drug interactions with SJW is presented in Table 2.

The photosensitizing effect of St. John’s wort is also well known, which reasonably contraindicates the use of this type of preparation in summer, during high sunlight. On the other hand, the photosensitizing effect of hypericin provides a background for the use of this compound in photodynamic therapy [114,115]. As a side note, all these indications regarding the safety of SJW preparations have been the reason why dietary supplements and OTC monopreparations containing relatively high doses of dry St. John’s wort extract (tablets/capsules containing 160–425 mg) have been withheld from the Polish pharmaceutical market.

Saffron is used in foods and is generally regarded as safe when consumed in usual quantities. Ingestion of less than 1.5 g of saffron is nontoxic for human, and it is considered toxic when ingested with doses more than 5 g. The estimated lethal dose is about 20 g/day [116]. The data indicate that the frequency and types of adverse events reported for saffron used as antidepressant are similar to those reported for placebo and standard antidepressants (fluoxetine, citalopram) used as comparators. Spontaneous reports of adverse reactions associated with saffron include rash, flushing, hyperhidrosis, vomiting, malaise and insomnia. However, it must be emphasized that causality has not necessarily been established in all these cases [117].

Other plant antidepressants are also characterized by high safety of use. Lemon balm is generally well tolerated, having no relevant side effects, and only occasionally headache, vomiting, abdominal pain and nausea have been reported [118]. Further, no significant adverse effects associated with the use of lavender preparations in usually appropriate doses have been described [119]. In general, ginkgo administered in antidepressant preparations is also safe and well tolerated. The maximum recommended dose for ginkgo extract is 240 mg/day [120]. The reported gingko-induced adverse effects were mild and included headache, heart palpitations, gastrointestinal upset, constipation and allergic skin reactions [121]. However, it should be emphasized that the biologically active ingredients of gingko are inhibitors of the cytochrome CYP2C9 (important for the metabolism of selected oral anticoagulants and antiplatelet drugs) and inducers of CYP2C19 (important for the metabolism of selected anticonvulsants). Therefore, patients treated with warfarin, diazepam or phenytoin should avoid gingko preparations due to the increased risk of bleeding or seizures, despite anticoagulant/anticonvulsant compliance [122]. Panax ginseng generally is well tolerated, and its adverse effects are mild and reversible and include nausea, diarrhoea, euphoria, insomnia, headaches, hypertension, hypotension, mastalgia and vaginal bleeding. However, it should be noted that biological compounds from Panax ginseng may interact with caffeine to cause hypertension, and it may decrease the effectiveness of warfarin. Concomitant use of Panax ginseng and the monoamine oxidase inhibitor phenelzine may result in manic-like symptoms. Ginseng also exerts hypoglycaemic activity; therefore, caution should be exercised in using ginseng products in patients with diabetes because of possible pharmacodynamic interactions with oral hypoglycaemic agents and insulin [123]. Roseroot is well tolerated, and characteristic adverse effects have not been described. Only a few reports have indicated that repeated doses of roseroot caused mild dizziness and gastrointestinal discomfort. However, it can be mildly stimulating for some people; therefore, taking roseroot late in the day should be avoided to prevent potential interference with sleep. Some sources suggest avoiding using roseroot in people with bipolar, hypomania or paranoia, and as a preventive measure, roseroot preparations should not be combined with coffee [124]. Moreover, the use of the main biologically active ingredients of magnolia bark (magnolol and honokiol) seems to be safe. No specific adverse effects have been described for these substances at a concentration of > 240 mg/kg b.w./day of magnolia bark extract. Intervention trials employing concentrated magnolia bark extract for up to 1 year did not report adverse effects. In conclusion, over the recent years, different food safety authorities evaluated magnolol and honokiol and considered them safe [125]. Data on the side effects of other plants discussed in this review are scarce, and the literature search does not indicate reporting significant disorders during their use. A Sayyah et al. study [126] did not demonstrate any significant differences between groups of patients treated with either 500 mg aqueous extract of borage or fluoxetine (20 mg/day). In a randomized, double-blind, placebo-controlled clinical study aiming to determine the effect of brahmi on attention, cognitive processing and working memory in healthy elderly, no significant adverse effects were demonstrated during the trial in subjects treated with brahmi extract tablets containing either 300 or 600 mg compared to the placebo group [127]. Mimosa tree is considered safe for long-term use. Aqueous extract of mimosa tree was not found to produce any delirious symptoms, and the plant is regarded to be safe even at the dose 2000 mg/kg p.o. [128].

## 8. A Brief Description of the Phytopharmacodynamics of Plant-Derived Compounds with Antidepressant Activity with Particular Emphasis on Their Anti-Inflammatory Effect

There are several plants usually administered in depression phytopharmacotherapy. In the opinion of the authors of this review and based on literature data [15,90,93,96], several medicinal plants with great potential and a history of use in depression phytopharmacotherapy can be identified. They are listed in Table 3. The authors use their common names in this paper.

Classical pharmacotherapy of depression is still based on the monoamine theory and aims to correct the disturbed CNS neurotransmitter levels. In general, the detailed mechanisms by which medicinal plants exert antidepressant effects do not differ from those demonstrated for classic, pharmacological antidepressants. The evidence for their phytopharmacodynamics comes mainly from experimental studies and literature data reported within traditional medical systems, and pharmacopoeias support the use of some herbs in the treatment of depression. The description of the antidepressant activity of selected plant-derived compounds involves several mechanisms, including inhibition of monoamine reuptake; enhanced serotonin receptor binding and sensitization; monoamine oxidase inhibition; GABAergic effects (especially for plants exhibiting sedative and anxiolytic effects accompanying the antidepressant effect); complex, excitatory or inhibitory effects on various receptors (N-methyl-D-aspartic acid (NMDA), GABA, cholinergic, adrenergic, serotonergic, dopaminergic and opioid ones); and cannabinoid system effects [15,129,130,131,132]. In line with the complex, psycho-neuro-immuno-endocrinological pathogenesis of depression, herbal compounds have also been found to affect the activity of the HPA axis and stimulate immunomodulatory activity, which seems to contribute significantly to their antidepressant effect. Considering the mechanism of action of medicinal plants with antidepressant activity, it should be noted once again that their antidepressant effect results from the comprehensive action of numerous active compounds (in line with the theory of polyvalence and synergistic action of plant-derived compounds mentioned above). Due to the complexity of the chemical composition of medicinal plants with antidepressant activity (the most important ingredients are listed in Table 4), the final effect depends on their synergistic action. Thus, unlike traditional synthetic antidepressants, the molecular mechanism of action of herbal preparations cannot be explained based on a separate analysis for individual compounds; instead, it is considered a result of the collective and simultaneous action of many active compounds co-occurring in the studied plant extract. In addition, possible differences in the composition of medicinal plants resulting from the plant sources (harvest from cultivation vs. from a natural stand) and seasonal fluctuations in the chemical composition of plants contribute to the difficulties in an unambiguous description of the phytopharmacodynamics of plant preparations. Moreover, although research on plant-based drugs provides an important source of new antidepressants, it faces numerous problems, including the procurement and authentication of plant material, implementation of high-throughput screening bioassays and scale-up of bioactive compounds with suspected antidepressant activity subjected to clinical assessment. The issues mentioned above pose a challenge to translational pharmacology and the detailed description of plant-derived preparations entering clinical trials [133,134].

**Figure 2:**
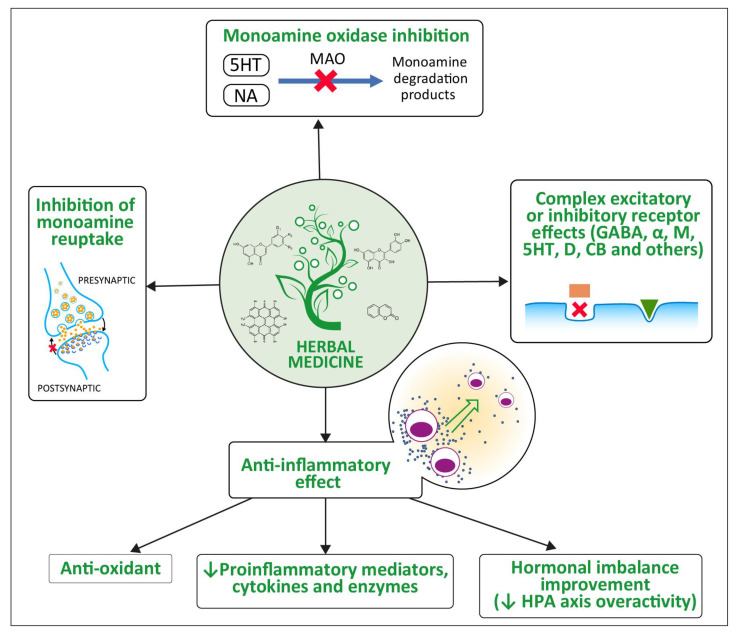
The summary of the elements of the mechanisms of action of plants with antidepressant activity. According to the theory of synergistic and polyvalent action of active ingredients contained in medicinal plants (symbolically marked in the figure with selected chemical formulas), the antidepressant effect is the result of the action of numerous co-occurring active compounds. The pharmacodynamics of these compounds involve similar mechanisms to those attributed to synthetic antidepressants. However, unlike synthetic antidepressants, an important aspect of the antidepressant effect of herbal preparations is also their anti-inflammatory action. Details are given in the text. (HPA—hypothalamic-pituitary-adrenal axis; 5HT—5-hydroxytryptamine; serotonin; NA—noradrenaline; GABA—gamma-aminobutyric acid; α—adrenergic; M—muscarinic; D—dopaminergic; CB—cannabinoid).

Figure 2 presents the essential elements of the phytopharmacodynamics of antidepressant medicinal plants.

In addition to the direct effect of active, plant-derived compounds on correcting the disturbances of CNS neurotransmission, an immunomodulatory effect is also considered important to their antidepressant activity. There is evidence that antidepressant plants discussed in this review exert anti-inflammatory effects, also involving CNS. The pathophysiology of depression, as mentioned in the brief description above, is also associated with immune disturbances, releasing pro-inflammatory mediators and increased oxidative stress in the CNS. Hence, the alleviation of immunological disturbances may contribute to an antidepressant effect. The anti-inflammatory effects of plants with antidepressant activity examined in this review are briefly discussed below.

Brahmi has been used for nearly 3000 years by Ayurvedic medical professionals for Alzheimer’s disease, improving memory, anxiety, allergic conditions and irritable bowel syndrome. It is a medicinal herb exerting an anti-inflammatory effect due to the selective inhibition of the cyclooxygenase-2 (COX-2) enzyme. Therefore, it is used in relieving acute pain and inflammation due to a reduction in COX-2-mediated prostanoid mediators. In addition, brahmi helps manage diseases involving chronic systemic and brain inflammation driven by the innate immune system. The administration of brahmi is associated with cognitive enhancing (nootropic) activity, including improving free recall, observed after prolonged intake (>3 months) due to the alleviation of chronic inflammation and oxidative stress associated with ageing. Furthermore, brahmi use is associated with the down-regulation of NO and pro-inflammatory cytokines: TNF-a and Il-6, and elevation of Il-10 in stimulated human blood cells [135,136]. Moreover, an additional element of the anti-inflammatory action of brahmi in the brain is the inhibition of signalling enzymes associated with CNS inflammatory pathways: caspase-1 and matrix metalloproteinase-3, as well as caspase-3, which has been shown to cleave protein tau, an early event in the development of Alzheimer’s disease [136]. The brahmi extract solution demonstrated antioxidant activity in the 2,2-diphenyl-1-picrylhydrazyl (DPPH) radical scavenging method [137]. It was also shown in an experimental study that brain antioxidant status improved in cigarette smoke-exposed rats treated with an extract from brahmi [138,139].

Current pharmacological studies show that borage has analgesic, anxiolytic, antibacterial and antiviral properties. A decoction and hydroalcoholic extracts of borage showed promising antioxidant activity evaluated by DPPH and 2,2′-azino-bis(3-ethylbenzothiazoline-6-sulphonic acid (ABTS) assays, which are commonly applied to determine total antioxidative potential [140]. Borage also shows anti-inflammatory properties. An in vitro study revealed that macrophages treated with a borage hexane extract modulated their inflammatory mode by reducing NO secretion and COX-2 activity and decreasing IL-1β, IL-6 and TNF-α cytokine levels [141].

Ginkgo is another medicinal plant with antidepressant potential. However, it also has anticancer, antidementia, antidiabetic, antiobesity, antilipidemic, antimicrobial, antiplatelet, hepatoprotective, anti-ageing and neuroprotective effects. It is frequently employed to treat neurological, cardiovascular and respiratory diseases, including tardive dyskinesia [142]. This plant also offers immunomodulatory and anti-inflammatory properties. An experimental study evaluated the protective potential of ginkgo extract against hippocampal neuronal injury induced by trimethyltin (TMT). A significant decrease in oxidative stress, as evidenced by reductions in malondialdehyde (MDA) and total reactive oxygen species (ROS) and marked suppression of nuclear factor kappa-light-chain-enhancer of activated B cells (NF-κB) and pro-inflammatory cytokines (TNF-α, IL-1α, 1L-6), was demonstrated in rats treated with the ginkgo extract [143]. In another experimental in vitro study using lipopolysaccharide (LPS) treated cultured primary rat microglia, the ginkgo extract significantly inhibited the release of prostaglandin E2 (PGE2) and differentially regulated pro-inflammatory cytokines (TNF-α, IL-6 and IL-1β). Thus, it can be concluded that ginkgo showed anti-neuroinflammatory activity [144]. In macrophage culture, the ethanol extract of ginkgo flowers and the chloroform and ethyl acetate fractions significantly decreased nitric oxide (NO), interleukin-6 (IL-6) and PGE2 production [145]. Ethanol and acetone extracts from ginkgo added into the culture of human endothelial cells also inhibited ROS production and decreased soluble intercellular adhesion molecule-1 (ICAM-1), vascular cell adhesion molecule-1 (VCAM-1) and E-selectin adhesion molecule levels [146].

Another medicinal plant used in the additional treatment of neurodegenerative diseases, cardiovascular disease, hypertension, insulin resistance, cancer and other degenerative processes commonly developing with age is Korean ginseng. The administration of preparations from this plant offers several health benefits related to anti-inflammatory and decreasing oxidative stress effects associated with ageing. Korean ginseng bioactive compounds reduce the effects of these conditions, mainly due to the suppression of the COX-2 and 5-lipoxygenase (5-LOX) enzymes. They can also decrease the production of malonaldehyde and increase the expression of antioxidants (glutathione and superoxide dismutase). Furthermore, the chronic administration of preparations from Korean ginseng resulted in the down-regulation of TNF-α, IL-1*b* and IL-6. Active compounds from Korean ginseng also caused an increase in cellular proliferation; an increase in the activity of free radical scavengers; and the activation of extracellular signal-regulated kinases, mitogen-activated protein kinase (MAPK) pathways and hypoxia-inducible factor 1-alpha (HIF-1*a*) [147,148]. In vitro studies also reported ginseng saponins as NO synthesis inhibitors in LPS- and IFN-γ-induced murine microglial cells [149].

Lavender is used for restlessness, insomnia, nervousness and depression. It is also administered for various digestive complaints, including dyspepsia, loss of appetite, vomiting and nausea. Lavender essential oils were also studied in macrophage cell lines as an in vitro cell culture model for evaluation of its potential efficacy in LPS-stimulated inflammation. It was demonstrated that compounds constituting the lavender essential oil modulate the activity and action of the NF-κB signalling pathway and are potent inhibitors of the synthesis of four pro-inflammatory cytokines: IL-6, IL-8, IL-β and TNFα [150]. The anti-inflammatory activity of lavender oil was also revealed in an animal study of inflammation induced by carrageenan and croton oil. This inflammation model shows increased cytokine, prostaglandin and leukotriene production. These effects are thought to be mediated by protein kinase C, which mediates a number of intracellular signal transduction pathways implicated in the pathogenesis of inflammation, including phospholipase A2-dependent arachidonic acid release and eicosanoid production. Animals pretreated with lavender oil demonstrated decreased inflammatory response [151]. In an acute model of inflammation (carrageenan-induced paw oedema model) in mice, myeloperoxidase (MPO) activity and NO production were decreased in animals treated with lavender essential oil [152].

Lemon balm is another popular herb with multiple therapeutic properties, including antidepressive, antispasmodic and antimicrobial effects. This medicinal plant reduces stress and anxiety and promotes sleep. Moreover, lemon balm has marked anti-inflammatory and antioxidant properties. This plant is also used to treat neurodegenerative diseases and obesity. Additionally, it finds application in ophthalmology, gynaecology, oncology, gastroenterology and cardiology [153,154]. In an animal study, in the carrageenan paw oedema model in rats, an antioxidant capacity of lemon balm extract was demonstrated, including the ability to scavenge a wide range of free radicals, including nitric oxide. The mechanisms of antioxidant action of lemon balm extract involve improving plasma levels of catalase, superoxide dismutase and glutathione peroxidase, as well as a marked reduction in plasma DNA damage, myeloperoxidase and lipid peroxidation [155]. Noteworthy, essential oils from lemon balm are also a rich source of phenolic antioxidants (mainly citronellal and neral), and its activity is comparable with synthetic antioxidants: butylated hydroxyanisole (BHA) and butylated hydroxytoluene (BHT) [155]. The anti-inflammatory action of lemon balm, similar to other medicinal plants discussed in this review, was revealed to be attributed to the alleviation of reactions induced by prostaglandins and some pro-inflammatory (TNF-α, IL-1 and IL-6) cytokines [154].

Magnolia bark also shows a strong anti-inflammatory effect. This medicinal plant has been used for thousands of years in Chinese and Japanese medicines to treat anxiety, asthma, depression, gastrointestinal disorders and headache. The main compounds with anti-inflammatory effects are honokiol and magnolol. Honokiol inhibits the TNF-α-stimulated NF-κB pathway, with subsequent inhibition of NO generation. Moreover, honokiol reduces NF-κB target genes, such as VEGF, ICAM-1 and COX-2. This compound is regarded as a potent inhibitor of ROS, with estimated antioxidant activity 1000 times that of α-tocopherol (vitamin E) [156]. In an experimental in vitro study, magnolia bark extract reduced matrix metalloproteinase 2 (MMP-2) and matrix metalloproteinase 9 (MMP-9) secretion from LPS-stimulated monocytes [157]. Both honokiol and magnolol have antioxidant properties [158]. Honokiol significantly inhibited the LPS-induced TNF-α synthesis and NF-κB activity in mouse monocytes [159].

Mimosa tree is a medicinal plant with antidepressant, anticancer, antibacterial, antiallergic, antinociceptive, hepatoprotective, antidiabetic, anti-inflammatory and antioxidant effects [160,161]. Similar to other medicinal plants mentioned above, the mimosa tree also exerted an anti-inflammatory effect in experimental carrageenan, dextran and cotton pellet-induced rat models of inflammation and paw oedema [162]. Furthermore, in the chronic rat model of inflammation, the aqueous extract of mimosa tree alleviated both the first phase of the inflammatory response produced by histamine, serotonin, prostaglandins and bradykinin and inhibited the second transudative and proliferative phase associated with cyclooxygenase products of the entity [163,164]. Again, as with the other medicinal plants discussed above, the anti-inflammatory effect of mimosa tree is accompanied by an antioxidant effect due to potent free radical scavenging effects comparable to those of ascorbic acid and activation of superoxide dismutases and glutathione peroxidase catalase [161,165].

An anti-inflammatory effect is also reported for roseroot, and this action is conditioned by specific compounds: salidroside and rosavin. Roseroot is considered an adaptogen—it means that the plant stimulates the body’s resistance to physical, environmental and emotional stressors. Thus, it is used to fight fatigue, anxiety, stress and depression. The anti-inflammatory property of extracts from this medicinal plant finds use in various pathological conditions, including cardiovascular disease, neurodegenerative diseases, metabolic disease, arthritis or cancer [166]. Both in vitro and in vivo experiments confirmed the immune-regulation effects of roseroot extract via various inflammatory mediators (e.g., TNFα, IL-6, IL-1β, NO, COX-2) and signalling pathways (NF-κB, activator protein 1 (AP-1) and signal transducer and activator of transcription 3 (STAT3)) [166,167]. Furthermore, an experimental study confirmed the anti-inflammatory and neuroprotective effects of roseroot constituents in microglial and neuronal cells. Activated microglia produce large amounts of reactive oxygen species, nitric oxide and pro-inflammatory cytokines, such as TNF-α, interleukin-1β (IL-1β) and interleukin-6 (IL-6), which, in turn, cause neuronal damage. Moreover, the active compound of roseroot protects against glutamate-induced nephrotoxicity. Thus, roseroot preparations may offer some health benefits in neurodegenerative disorders [168].

A pronounced immunoregulatory effect is also documented for dried stigmas of the saffron crocus. This plant may have the potential to treat cancer and age-related macular degeneration. However, it has a well-documented efficacy as an alternative treatment for mild to moderate depression. The putative anti-inflammatory action of saffron crocus is likely caused by crocin, crocetin and safranal. The molecular mechanisms of these derivatives involve a decrease of serum levels of NF-κB p65 subunit, TNF-α, IFN-γ and some interleukins, such as IL-1β, IL-6, IL-12 and IL-17A. Moreover, saffron crocus has been known as the antagonist of NF-κB and the agonist of peroxisome proliferator-activated receptor gamma (PPAR-γ). In addition, this flower was shown to down-regulate pro-inflammatory enzymes, such as MPO, COX-2, inducible nitric oxide synthase (iNOS) and phospholipase A2, inhibiting prostanoids synthesis [169]. In a clinical study of patients with type 2 diabetes, 12 weeks of supplementation with saffron tablets (100 mg/day) yielded no significant differences between groups treated with saffron crocus and placebo regarding TNF-α, but the supplementation resulted in a marked decrease in blood MDA level, which is a marker of oxidative stress [170]. Similarly, patients with chronic obstructive pulmonary disease supplemented with saffron crocus (30 mg/day of crocin during 12 weeks) demonstrated decreased serum levels of total oxidative status and NF-κB, which indicated that saffron supplementation appears to effectively establish oxidant/antioxidant balance and improve inflammatory conditions in patients with COPD [171]. The anti-inflammatory property of saffron crocus was also proved in asthma patients. The 8-week administration of 100 mg/day of saffron crocus preparation resulted in a significant increase of IL-10, IL-35 and transforming growth factor beta (TGF-β) [172]. Some experimental studies showed that saffron crocus contributes to neuroprotection. The saffron crocus compounds decrease CNS inflammation by inhibiting the production of free radicals and enhancing antioxidant activities in the extracellular signal-related kinases 1 and 2 (ERK1/2) pathway-dependent manner. Moreover, saffron crocus preparations enhance gamma-glutamylcysteine synthase activity, the main enzyme for glutathione synthesis [173].

St. John’s wort is one of the most popular medicinal plants. It has been used in digestive disorders, e.g., dyspepsia and spastic ailments of the digestive tract (for the relaxation of the smooth muscles of the digestive tract and bile ducts). It also has cholagogic and cholepoietic effects. Among the numerous biological properties of SJW, the anti-inflammatory effect should be stressed. The medicinal plant has a long history of traditional use in inflammatory conditions, e.g., neuralgia, fibrositis, rheumatism and sciatica. It is also applied externally to treat wounds and bruises [174]. A key anti-inflammatory mechanism of SJW is the inhibition of the expression of pro-inflammatory genes, including COX-2, IL-6 and iNOS [175]. PGE2 is formed from arachidonic acid (AA) by cyclooxygenase-catalysed synthesis of prostaglandin H2 (PGH2) and further transformation by PGE2 synthases. Experimental studies demonstrated that one of the main compounds of SJW, hyperforin, potently inhibited the enzymatic conversion of PGH2 to PGE2, catalysed by PGE2 synthases. Moreover, hyperforin was also found to inhibit 5-LOX. It also contributes to the anti-inflammatory and anti-cancerogenic properties of SJW [176]. In the context of the anti-inflammatory effect of St. John’s wort within the CNS and the associated antidepressant effect, experimental studies have shown that mouse hippocampal neurons were protected against glutamate- or NMDA-induced cytotoxicity by SJW extract. Moreover, a morphological remodelling by increasing neurite outgrowth and activation of the anti-inflammatory defence by inhibiting cytokine production was reported in human macrophages in the presence of SJW extract. These neuroprotective properties may be the beneficial antidepressive effect of SJW supplementation [177].

In conclusion, the evidence discussed briefly above confirms the anti-inflammatory properties of medicinal plants showing antidepressant activity. In the context of the complex pathogenesis of depression, which also includes immunological disturbances within the brain, it should be emphasized that the anti-inflammatory potential of the medicinal plants discussed in this review is an important element of their antidepressant activity. Details on the mechanisms of antidepressant action of individual plants discussed here are presented in Table 4.

In addition, the complex antidepressant effect caused by phytopharmacologically active ingredients is often accompanied by an anxiolytic effect, which alleviates sleep disorders, improves cognitive function and counteracts adynamia and fatigue. This is due to the complex action of phytopharmacological compounds. Further, the secondary anxiolytic effect induced by herbal antidepressants may be due to a “halo effect”, which means that anxiety may also be reduced if depression is successfully treated [15,178]. It should be stressed once again that the mechanisms of action of active ingredients present in medicinal plants with antidepressant properties, in accordance with the principles of synergy and polyvalence, are not as clearly defined as for synthetic, single antidepressants.

Table 4 lists plants considered to show antidepressant activity, with details regarding their mechanisms of antidepressant action.

The literature review also revealed the results of some clinical trials evaluating the efficacy of the antidepressant medicinal plants discussed in this paper. Although the number of these studies is much smaller than studies evaluating synthetic antidepressants, and they are subject to some caveats, as discussed below, the available data support the efficacy of phytopharmacotherapy in mild to moderate depression, with an emphasis on the lower potential for adverse effects. In line with the principles of evidence-based medicine (EBM), Table 5 presents data obtained from the highest level of scientific evidence (systematic reviews, meta-analyses and isolated, methodologically correct (randomized, blinded) clinical trials) [232,233,234]. For some plants (bacopa, mimosa tree, magnolia), the literature review revealed a clear advantage of experimental animal studies and no results from more extensive clinical trials. In these cases, we also enrolled prospective, observational studies. This further justifies the need to undertake large clinical trials evaluating the potential antidepressant efficacy and safety of these plants in patients with depression. Researchers also emphasize that the vast majority of clinical trials conducted to date evaluating herbal antidepressants have numerous limitations. We should interpret the results of these studies with caution due to the high level of heterogeneity between them. The small sample sizes, relatively small follow-up period and differences between the detailed methodology were the most listed limitations of the present clinical trials. Some of these were not randomized, double-blind, placebo-controlled trials, which may lead to potential selection bias and may not exclude natural improvement. Therefore, further high-quality clinical trials are needed to firmly establish the clinical efficacy of medicinal plants with antidepressant effects

## 9. Conclusions and Future Research Directions

Many medicinal plants exert a range of psychotherapeutic effects through their influence on central nervous system activity, including antidepressant, anxiolytic, sedative, hypnotic or cognitive effects. Moreover, medicinal plants with adaptogenic and toning effects are important in phytopharmacotherapy because they are believed to enhance adaptation to exogenous stressors through complex and pleiotropic neuroendocrine mechanisms [261,262]. The discussion in this review indicates the pharmacological effectiveness of phytotherapy in correcting pathophysiological disturbances and alleviating the symptoms of depression. The mechanisms of action of herbal-derived active compounds with antidepressant activity presented in this narrative review confirm similar pharmacodynamics to synthetic antidepressants. In addition, a literature review yielded some scientific evidence (systematic reviews, meta-analyses and randomized controlled clinical trials) that indicates the clinical effectiveness of the medicinal plants discussed in this paper in treating mild and moderate depression. It makes phytopharmacotherapy a valuable alternative to classical antidepressant treatment (SSRIs). Antidepressant phytotherapy involves a lower risk of side effects. However, one should not forget that it is not entirely devoid of them, which has been particularly demonstrated for St. John’s wort preparations.

According to the authors, taking into account the number of studies carried out so far, the greatest clinical experience regarding the use of phytopharmacotherapy in depression should be attributed to St. John’s wort and Saffron preparations. However, we should emphasize that there are some limitations concerning the methodological quality of clinical studies evaluating the phytotherapy of depression. These concerns necessitate further verification of the antidepressant effect of medicinal plants in large, appropriately designed clinical trials to yield conclusive and incontrovertible results confirming the efficacy and safety of herbal antidepressants.

In addition to the medicinal plants described in this review, other plants are currently being studied for their antidepressant activity. Examples of such plants include *Asparagus racemosus*, *Rosmarinus officinalis*, *Curcuma longa*, *Camellia sinensis*, *Emblica officinalis*, *Cucurbita pepo*, *Centella asiatica*, *Glycyrrhiza glabra*, *Piper methysticum* and others [3,4,263,264]. Antidepressant activity is also sought in plants with documented sedative, hypnotic and anxiolytic properties, such as *Humulus lupulus* (hops), *Valeriana officinalis* (valerian) and *Passiflora incarnata* (maypop) [192]. A promising direction in the search for new antidepressant plants may also be turning to traditional Chinese and Indian medicine systems. Evidence is being reported that plants exotic to Europeans, found in Africa or South America, or those widely used for centuries in the traditional folk medicine of East Asia (China, Japan) also have antidepressant potential. The prophylaxis and adjuvant treatment of depression were demonstrated for the genera *Aloysia*, *Gladiolus*, *Hemerocallis* or *Convolvulus,* commonly used in Ayurvedic and folk medicine. *Aloysia virgata* grows in Brazil, Bolivia, Argentina and Paraguay. The plant is used as an anti-catarrhal, antirheumatic, diaphoretic, stimulant, stomachic and emollient. Plants from the *Aloysia* genus are also traditionally used for affective disorders, and some have proven anxiolytic and antidepressant activity. Animal studies using the tail suspension test (TST) and forced swimming test (FST) demonstrated promising antidepressive activity of the ethanolic extract of *Aloysia* [265].

*Hemerocallis citrina* is a plant indigenous to Asia. It is used in the folk medicine of East Asia (China, Japan) and North America to improve emotional health and treat various diseases, including insomnia, hepatosis and cancer [266]. Recent clinical studies confirm the sedative effects and high efficiency of ethanol extracts from the plant in mitigating sleep and memory disorders, with the antidepressant effect currently being studied in animal models [267]. *Gladiolus dalenii* is used by local communities in Africa to treat various infections, such as meningitis, malaria, diarrhoea, ulcers and HIV-related fungal infections [268]. In African ethnomedicine, especially in Cameroon, *Gladiolus* is regarded to be a cure for various CNS disorders, such as epilepsy, convulsions, schizophrenia and mood disorders. There are reasons to believe preparations from this plant show antidepressant efficacy in animal models of epilepsy. Animal experiments based on the TST and FST also showed the potential effectiveness of this plant in an animal model of depression [267]. *Convolvulus pluricaulis* (shankhpushpi) is one of the perennial medicinal herbs described in Ayurvedic literature. This plant is reported to improve memory skills as a psychostimulant with a calming effect, reducing mental tension [269]. Pharmacological studies indicate that compounds found in *Convolvulus pluricaulis* interact with various proteins, neurosynapses, signalling pathways and serotonergic synapses, which play a crucial role in the pathophysiology of Alzheimer’s disease and neurotransmission abnormalities related to long-term depression [270].

In general, according to Moragrega and Rios, there were about 650 reports of antidepressant-like medicinal plants in the PubMed database (considering the timespan from January 2000 to March 2020). There were 155 species studied and reported as antidepressants or as sources of active principles for treating this condition in preclinical studies [271,272]. A paradigm shift in current research should also be noted—the main research directions are shifting from the classic correction of neurotransmission to the immunological and inflammatory aspects, in accordance with the broad “psycho-neuro-immuno-endocrinological” pathophysiological concept of depression. Thus, current research focuses not only on verifying the effect of novel plant-derived preparations on CNS neurotransmission, but also confirming the phytopharmacological activity in the mitigation of pro-inflammatory mediators and enzymes [271,272]. Therefore, the novel herbal antidepressants expected in the near future will not strive to rectify monoamine disturbances in the CNS, but will focus more on immunoregulatory effects, targeting immunological and hormonal disorders, which so far have been secondary pathophysiological targets of pharmacotherapy not covered by classical treatment with synthetic antidepressants.

## Figures and Tables

**Table 1:** Selected antidepressants (OTC drugs or dietary supplements) available in Poland.

Brand Name	Marketing Authorization Holder	Pharmaceutical Form	Active Ingredients (per Dose)	Dosage	Declared Effects by MAH
MIRALO	USP Zdrowie, Warszawa	Capsules	100 mg *Withania somnifera* (ashwagandha) extract (7 mg withanolides), 28 mg saffron stigma extract (0.84 mg crocin and 0.56 mg safranal), 100 mg lemon balm extract (2 mg rosemary acid)	1 capsule a day	Improves mood, helps maintain a state of relaxation, contributes to emotional balance.
JASNUM MOOD	USP Zdrowie, Warszawa	Capsules	30 mg saffron stigma extract, vitamin B6 (1.4 mg), vitamin B12 (100 μg)	1 capsule a day	Alleviates hormonal disorders during menopause that cause mood swings.
DEPRIBON	Bonimed, Żywiec	Capsules	150 mg of St. John’s wort extract, 50 mg of Ginseng root extract	1 capsule a day	Improves mood.
INTRACTUM HYPERICI	Phyto Pharm, Klęka	Liquid	Ethanol extract of *Hypericum perforatum*L., *herba* (fresh St. John’s wort) *Hyperici herbae recentis intractum* (1:1). Extraction solvent: ethanol 96% (*v*/*v*)	4 times a day, 5 mL of the preparation in a small amount of liquid. The recommended treatment time is 4 weeks.	Traditional herbal medicine used to relieve temporary states of nervous exhaustion. It also relieves depression.
DEPRESANUM	Novascon, Warszawa	Coated tablets	Inositol 100 mg, L-tryptophan 100 mg, saffron flower extract 15 mg, vitamin B6 0.210 mg, folic acid 100 μg	1 tablet 2 times a day during a meal	Helps maintain a positive mood, reduces fatigue and tiredness, improves nervous system function.
SZAFRACEUM	Lekam, Warszawa	Tablets	Saffron extract 30 mg, inositol 100 mg, L-tryptophan 50 mg, ginseng root extract 50 mg, vitamin B6 1.3 mg, vitamin B2 1.3 mg, vitamin B12 2.5 µg, folic acid 200 µg	1 tablet a day	Helps maintain a positive mood, supports the maintenance of emotional balance.
DEPRELLA	Organic Pharma, Warszawa	Capsules	L-tryptophan 150 mg, L-phenylalanine 150 mg, turmeric extract (*Curcuma longa*) 10:1–100 mg, saffron crocus extract 30 mg, vitamin D (cholecalciferol) 5 µg, vitamin B6 (pyridoxine hydrochloride) 1.4 mg, vitamin B9 (pteroylmonoglutamic acid) 200 µg, vitamin B12 (cyanocobalamin) 2.5 µg	1 capsule a day	Improves mood and has an antidepressant effect by using the bio-potential of a harmonious combination of ingredients that support the production of neurotransmitters and happiness hormones and the transmission of nerve impulses. The composition is enriched with the addition of B vitamins, essential for the proper functioning of the nervous system and the maintenance of mental balance.
ALINESS	MedicaLine, Karczew	Tablets	Cultivated saffron crocus extract (*Crocus sativus*) SafraSOL 30 mg, including crocin 3 mg, safranal 600 µg	1–3 tablets a day during or after a meal with water	Improves mood, helps maintain emotional balance, improves libido, helps maintain comfort before and during the menstrual cycle.
HYPERHERBA	Labofarm, Starogard Gdański	Tablets	30 mg *Hypericum perforatum* L., *herba* (St. John’s wort)	1 tablet 3 times a day with water	Traditionally used to relieve temporary symptoms of mental exhaustion.
DEPREMIN	Colfarm, Mielec	Coated tablets	612 mg of extract (in the form of a dry, quantified extract) from *Hypericum perforatum* L., *herba* (St. John’s wort), which corresponds to: 0.6 mg–1.8 mg of total hypericin expressed as hypericin, 36.72 mg–91.80 mg of total flavonoids per rutin, not more than 36.72 mg of hyperforin Extraction solvent: ethanol 60% (*v*/*v*)	1 tablet a day	Used in the short-term treatment of mild depressive disorders.
MAGNOLIAMAX	K2Pharm, s.r.o. Opava, Czech Republic	Coated tablets	350 mg of magnolia bark extract 6% (21 mg honokiol), black pepper extract—5 mg	1 tablet a day	Supports restful sleep and helps maintain a good mood. Relieves fatigue and stress.

**Table 2:** Clinically significant interactions of St. John’s wort with drugs.

Drugs	Possible Mechanism of Interaction	Effect of Interaction on Drug
*Pharmacokinetic interactions*		
HIV protease inhibitors (indinavir, nelfinavir, ritonavir and saquinavir)	Induction of CYP3A4	Reduced blood concentrations with possible loss of HIV suppression
HIV non-nucleoside reverse transcriptase inhibitors (efavirenz, nevirapine)	Induction of CYP3A4	Reduced blood concentrations with possible loss of HIV suppression
Anticonvulsants (carbamazepine, phenobarbitone and phenytoin)	Induction of CYP3A4	Reduced blood concentrations with risk of seizures
Oral contraceptives	Induction of CYP3A4 and CYP1A2	Reduced blood concentrations with risk of unintended pregnancy and breakthrough bleeding
Oral anticoagulant-vitamin K antagonists (warfarin and phenprocoumon)	Induction of CYP2C9	Reduced anticoagulant effect and need for increased dose
Cyclosporin	Induction of CYP3A4 and the transport protein P-glycoprotein	Reduced blood concentrations with risk of transplant rejection
Theophylline	Induction of CYP1A2	Reduced blood concentrations and possible loss of control of asthma or chronic obstructive pulmonary disease
Digoxin	Induction of transport protein P-glycoprotein	Reduced blood concentrations and possible loss of control of heart rhythm or heart failure
*Pharmacodynamic interactions*		
SSRI (citalopram, fluoxetine, fluvoxamine, paroxetine and sertraline)	Increasing of serotonin concentrations	Increased serotonergic effects with risk of increased incidence of serotonin syndrome
Serotonin 5HT 1B/1D agonists (“Triptans”; (sumatriptan, naratriptan, Rizatriptan and zolmitriptan)	Increasing of serotonin concentrations	Increased serotonergic effects with risk of increased incidence of serotonin syndrome

**Table 3:** Latin and common names of medicinal plants whose antidepressant properties are discussed in this review (in alphabetical order).

Scientific (Latin) Name	Common Name
*Albizia julibrissin*	Mimosa tree
*Bacopa monnieri*	Brahmi water hyssop
*Crocus sativus*	Saffron crocus
*Echium amoenum*	Borage
*Ginkgo biloba*	Gingko maidenhair tree
*Hypericum perforatum*	St. John’s wort (SJW)
*Lavandula officinalis*	Lavender
*Magnolia officinalis*	Magnolia bark houpu magnolia
*Melissa officinalis*	Lemon balm common balm balm mint
*Panax ginseng*	Korean ginseng
*Rhodiola rosea*	Roseroot golden root

**Table 4:** Herbal antidepressants and their mechanisms of action (phytopharmacodynamics).

Medicinal Plant and Its Commonly Used Parts	Major Active Constituents	Mechanism of Action	Additional Effects on the Nervous System	References
**St. John’s wort** flowers leaves	- Naphthodianthrones: hypericin, pseudohypericin - Flavonoids: rutin, hyperoside, quercetin, isoqercetin - Phloroglucinols: hyperforin, adhyperforin	- Inhibition of monoamine oxidase-A (MAO-A) and -B (MAO-B) activity - Inhibition of the synaptosomal uptake of serotonin, dopamine and noradrenaline (with approximately equal affinity) - Significant affinity for adenosine, GABA(A), GABA(B) and glutamate receptors - Down-regulation of beta-adrenergic receptors and an up-regulation of serotonin 5 HT(2) receptors - Regulation of genes that control hypothalamic-pituitary-adrenal axis function	Neuroprotective; Improves cognitive functions	Composition: [179,180] Mechanisms: [181,182,183]
**Saffron crocus** dried thread-like parts of the flower (stigmas)	- Carotenoids: crocin, crocetin, picrocrocin - Volatile aldehyde: safranal - Flavonoids: kaempferol - Monoterpenes - Phenolic acids: hydroxycinnamic acid, hydroxybenzoic acid, chlorogenic acid, caffeic acid, methylparaben, gallic acid and pyrogallol	- Decreasing acetylcholinesterase activity in cerebral tissues - Inhibition of monoamine oxidase type A and B - Inhibition of the reuptake of monoamines (dopamine, serotonin, noradrenaline) - Glutaminergic NMDA receptor antagonism - Improving brain-derived neurotrophic factor signalling - Reduction of hypothalamic–pituitary–adrenal axis overactivity with elevated cortisol levels - Anti-inflammatory and antioxidant potential (reduction of multiple pro-inflammatory mediators: decreased mRNA expression of TNF-α, IL-1β, IL-6, IFN-γ, NF-κB, COX-2 and iNOS	Anxiolytic Anti-inflammatory	Composition: [184,185] Mechanisms: [186,187,188]
**Lemon balm** leaves	- Essential oil–volatile compound –geranial, neral, citronellal and geraniol - Triterpenes: ursolic acid and oleanolic acid - Phenolic acids: rosmarinic acid, caffeic acid and chlorogenic acid - Flavonoids: quercetin, rhamnocitrin and luteolin	- Cholinergic nicotinic and muscarinic receptor binding - Inhibition of GABA transaminase activity, leading to increased levels of GABA - Binding to GABA A receptors - Inhibition of monoamine oxidase-A (MAO-A) activity - Down-regulation of the turnover of 5-HT in the brain regions associated with serotonergic neurotransmission	Anxiolytic Sedative Neuroprotective	Composition: [189,190] Mechanisms: [191,192,193]
**Lavender** flowers	- Essential oil-volatile compounds: linalool, linalyl acetate, lavandulol, geraniol, bornyl acetate, borneol, terpineol and eucalyptol, lavandulyl acetate - Anthocyanins - Herniarin, Coumarin - Phytosterols - Tannins - Coumaric acid, Glycolic acid, Valeric acid, Ursolic acid,	- Affinity for the glutamate NMDA receptors - Binding to the serotonin transporter (SERT) - No affinity for GABA-A receptors - Impact on ionic conductance in neurons - Increasing BDNF and tropomyosin receptor kinase B (TrkB) levels in the hippocampus - Reduction of the non-selectively calcium influx through several different types of voltage-operated calcium channels (VOCCs) such as the N-type, P/Q-type and T-type VOCCs	Anxiolytic Sedative	Composition: [194,195] Mechanisms: [196,197,198]
**Roseroot** root rhizomes	- Catechins, - Phenylpropanoids, phenylethanoids, phenolic acids (tyrosol, salidroside, trans-cinnamic alcohol derivatives-rosavin, rosarin, rosin)	- Inhibition of monoamine oxidase A (MAO-A) and B (MAO-B) activity - Activation of serotonin, dopamine and acetylcholine receptors with modulation of the release of many neurotransmitters, including glutamate, GABA and many hormones, including oxytocin, prolactin, vasopressin, cortisol, corticotropin, substance P - Modulation of the HPA axis activity - Stimulation of expression and release of neuropeptide Y (NPY) in neuroglial cells and up-regulation of heat shock protein Hsp-70 which, in turn, down-regulates - stress-induced JNK protein (suppressing glucocorticoid receptors and increasing cortisol) - Increase of beta-endorphins	Adaptogenic; Increases resistance to stress, reduces fatigue	Composition: [199,200] Mechanisms: [201,202,203]
**Ginkgo** leaves	- Flavonoids: kaempferol, quercetin - Terpenoids: ginkgolides Q, P, N, M, L, K, J, C, B and bilobalide - Alkylphenols - Lignans - Polysaccharides - Organic acids: ferulic acid, p-coumaric acid, protocatechuic acid, caffeic acid, p-hydroxybenzoic acid, m-hydroxybenzoic acid, vanillic acid, isovanillic acid, gallic acid, sinapic acid	- Antioxidant, anti-inflammatory and anti-apoptotic effects, which protect brain neurons and improve brain functions, including cognition - Inhibition of norepinephrine (NET), serotonin (SERT) and dopamine (DAT) uptake transporters and MAO activity - Modulation of cholinergic and monoamine pathways - Reduction of glutamate excitotoxicity	Neuroprotective; Improves cognitive functions	Composition: [142,204] Mechanisms: [205,206,207]
**Korean ginseng** root	- Ginseng triterpene saponins–ginsenosides: Rg1, Rc, Rd, Re, Rb1, Rb2, Rb0 - Gintonins (non-saponins): Ginseng major latex-like protein 151, Ginseng ribonuclease-like storage protein Lysophosphatidic acid - Organic acids - Aminoacids - Enzymes - Polysaccharides	- Regulating 5-HT_2A_ receptors - Inhibiting monoamine neurotransmitters reuptake - Modulating glucocorticoid receptor (GR) levels in both the prefrontal cortex (PFC) and hippocampus and decreasing HPA axis function - Improving BDNF expression in the hippocampus - Alleviation of neuroimmune system disturbances and inflammation within the brain: decreasing mRNA levels of IL-1b, IL-6, TNF-α, COX-2 and iNOS in the hippocampus and amygdala	Adaptogenic; Increases resistance to stress, reduces fatigue, improves cognitive functions	Composition: [208,209] Mechanisms: [210,211,212]
**Borage** leaves stems flowers	- Phenolics (pyrogallol, salicylic acid, gallic acid, caffeic acid) - Flavonoids (rutin, myricetin) - Fatty acids (γ-linolenic acid (GLA), α-linolenic acid (ALA), palmitic, stearidonic)-Anthocyanins - Organic acids: rosmarinic acid - Pyrrolizidine alkaloids - Enzymes: Δ-fatty acyl desaturase, Δ8-sphingolipid desaturase	- Reduction of neuroinflammation and oxidative stress: inhibition of the secretion of pro-inflammatory mediators (C-reactive protein) and cytokines (TNF-α, IL-1β, IL-6, IL-8) and blockade of the enzymatic activity of COX-2, lipoxygenase and iNOS - Increase in total antioxidant capacity of serum and brain tissue - Protective effect against oxidative damage to dopaminergic neurons by hydrogen peroxide - Reduction of NO levels in serum and brain tissue	Anxiolytic	Composition: [213,214] Mechanisms: [140,215,216]
**Brahmi** leaves stems	- Alkaloids: brahmin, nicotine, herpestine, bacosides A and B - Saponins A, B and C, triterpenoid saponins,-Phytosterols: stigmastanol, β-sitosterol, stigmasterol - Organic acids: betulinic acid, aspartic acid, glutamic acid, asiatic acid (ASTA) - α-alanine, serine - loliolide (LLD)	- Improvement of brain antioxidant activity - Noradrenergic activation - Regulation of mRNA translation and surface expression of neuroreceptors, such as AMPA, NMDA and GABA, in the various parts of the brain	Adaptogenic-increases resistance to stress, improves cognitive functions	Composition: [217,218] Mechanisms: [219,220,221]
**Mimosa tree** stem bark flowers	- Flavonoids: quercetin - Triterpene saponins: julibroside J29, julibroside J30, julibroside J31 - Macrocyclic alkaloids (budmunchiamines A, B and C)	- Regulation of the interaction between the monoamine system and BDNF, thereby, modulation the neuronal survival, neuroplasticity and neurogenesis - Inhibition of norepinephrine transporters and serotonin transporters - Activation of serotonergic systems, particularly the 5-HT_2A/2C_ receptors	Anxiolytic Improves sleep	Composition: [222,223] Mechanisms: [224,225,226]
**Magnolia bark** tree bark	- Biphenol compounds: magnolol and honokiol, obovatol - Alkaloids - Coumarins - Flavonoids - Lignans, Neolignans - Phenylpropanoid	- Mitigation of serotonergic and noradrenergic system dysfunction; significant increase in 5-HT and NE levels in the prefrontal cortex - Reduction in levels of the pro-inflammatory cytokines (TNF-α, IL-1β and IFN-γ) - Reduction of oxidative stress in neurons and microglia cells by inhibiting IFN-γ-mediated activation of NADPH oxidase - Enhancement of synaptic plasticity through activation of the hypoxia inducible factor (HIF-1α)-vascular endothelial growth factor (VEGF) signalling pathway in vitro and in vivo, as well as increased expression levels of synaptic protein 1 (SYN 1) and postsynaptic density protein 95 (PSD 95)	Anxiolytic	Composition: [227,228] Mechanisms: [229,230,231]

**Table 5:** Clinical evaluation of antidepressant activity—selected evidence from systematic reviews, meta-analyses and individual clinical trials.

Author, Year, References	Type of Publication	Characteristic	Conclusions
**St. John’s wort**			
Cui Y.H. et al. 2016 [235]	A meta-analysis	27 clinical trials (3126 patients). Evaluation of the efficacy and safety of SJW extract and selective serotonin reuptake inhibitors (SSRIs) in the treatment of depression.	SJW extract did not differ from SSRIs in clinical response, remission and the mean reduction in Hamilton Rating Scale for Depression score. St. John’s wort extract had a significantly lower rate of adverse events than SSRIs. SJW extract and SSRIs effectively treat mild-to-moderate depression.
Apaydin E.A. et al. 2016 [236]	A systematic review	35 clinical trials (6993 patients). Evaluation of the efficacy and safety of SJW in adults with major depressive disorder (MDD) compared to placebo and active comparator and evaluation of whether the effects vary by severity of MDD.	Compared to antidepressants, participants taking SJW were less likely to experience adverse events, with no difference in treatment effectiveness in mild and moderate depression. Monotherapy with SJW for mild and moderate depression was superior to placebo in reducing depression symptoms and not significantly different from antidepressant medication.
Ng X.N. et al. 2017 [237]	A meta-analysis	27 clinical trials (3808 patients). Evaluation of SJW efficacy and comparison to SSRIs.	SJW showed comparable response and remission in patients with depression and a significantly lower withdrawal/depression rate compared to standard SSRIs.
**Saffron crocus**			
Hausenblas H.A. et al. 2015 [238]	A systematic review	12 clinical trials (928 patients). The evaluation of scientific evidence from randomized controlled trials (RCTs) regarding the efficacy of saffron crocus on psychological and behavioural outcomes.	Saffron crocus can reduce the symptoms and effects of depression, premenstrual syndrome, sexual dysfunction and infertility.
Hausenblas H.A. et al. 2013 [239]	A meta-analysis	5 clinical trials (177 patients). A review of the clinical trials examining the effects of saffron crocus on the treatment of major depressive disorders (MDDs).	Saffron supplementation can reduce symptoms of depression in adults with MDD.
Jackson P.A. et al. 2021 [240]	A randomized, double-blind, parallel group clinical trial Clinical trial number: NCT03639831	The study assessed the efficacy of 8 weeks’ supplementation with 30 mg standardized saffron extract or placebo for 8 weeks on emotional well-being in 56 healthy males and females aged 18–54 years with subclinical feelings of low mood and anxiety and/or stress.	Participants who received the saffron crocus extract reported reduced depression scores and improved social relationships at the end of the study. This extract appears to improve subclinical depressive symptoms in healthy individuals and may contribute to increased resilience against the development of stress-related psychiatric disorders.
Akhondzadeh S. et al. 2020 [241]	A placebo-controlled, double-blind, randomized clinical trial	The study evaluated the effects of 30 mg of *Crocus sativus* capsules administered for 12 weeks on food craving, body weight and depression among 73 overweight (BMI ≥ 25) women with mild and moderate depression compared to placebo.	Saffron crocus capsules were not effective in reducing food cravings, but as a safe over-the-counter supplement, they were effective in reducing the symptoms of depression in patients who experience mild or moderate depression and are overweight.
**Lemon balm**			
Ghazizadeh J. et al. 2021 [242]	A systemic review and meta-analysis	Investigation of the effects and side effects of lemon balm as a medicinal herb on anxiety and depression in clinical trials published up to 30 October 2020.	Lemon balm significantly improved mean anxiety and depression scores compared with the placebo without serious side effects. Current evidence suggests that lemon balm may be effective in improving anxiety and depressive symptoms, particularly in the acute setting.
Haybar H. et al. 2018 [243]	A double-blind, randomized, placebo-controlled clinical trial	The study aimed to determine the effects of lemon balm supplementation (3 g during 8 weeks) on depression, anxiety, stress, and sleep disturbances in patients with chronic stable angina (CSA).	The intervention group receiving 8-week supplementation with 3 g lemon balm capsules had a significant reduction in scores of depression, anxiety, stress and total sleep disturbance in patients with CSA, compared to the placebo group.
Heidari M. et al. 2016 [244]	A double-blind, randomized, placebo-controlled clinical trial	The study evaluated the effect of lemon balm (Melissa Officinalis; 500 mg three times daily over 7 days) on depression in patients after coronary artery bypass graft.	After the intervention, herbal balm decreased depression more in the intervention group compared to the placebo group. Lemon balm may reduce depression after a coronary artery bypass graft.
**Lavender**			
Firoozeei T.S. et al. 2021 [245]	A systematic review and meta-analysis	17 clinical trials (1859 patients). Determining the efficacy of lavender on depression severity by performing a systematic review and meta-analysis.	The results showed a significant efficacy of lavender in reducing depression scores compared to the control group. Subgroup analysis showed that the effect of lavender was slightly more pronounced in participants with a diagnosis of depression, while its effect was statistically significant in patients with other illnesses with associated depressive symptoms, and the oral route was the most effective route of administration.
Nategh M. et al. 2022 [246]	A single-blind, randomized clinical trial	The study aimed to investigate the effects of lavender essential oil (two drops of lavender essence dropped on non-absorbent three-ply tissue paper attached to the patient’s shirt collar) on anxiety and depression in 110 patients with the acute coronary syndrome (ACS).	The study confirmed the effectiveness of lavender aromatherapy in reducing anxiety and depression in ACS patients and supports the use of lavender oil aromatherapy by intensive care nurses as a non-pharmacological and cost-effective intervention to reduce psychological tension and increase patient satisfaction during hospitalization in cardiac care units.
Tayebi A. et al. 2015 [247]	A randomized controlled clinical trial	The study evaluated the effect of aromatherapy with lavender essential oil on depression, anxiety and stress rates in 60 haemodialysis patients who inhaled the lavender essential oil smeared on a piece of cloth (three drops of oil) for one hour during the haemodialysis procedure.	The administration of lavender oil decreased the scores of depression and stress in the intervention group in comparison with the control group. Still, the reduction in the anxiety score was not statistically significant. Aromatherapy with lavender essential oil might reduce depression and stress among haemodialysis patients. Therefore, this method can be used as a complementary method with less complication to improve the quality of life of these patients.
Jokar M. et al. 2020 [248]	A single-blind, placebo-controlled, randomized clinical trial	Evaluation of the effect of inhaled lavender aromatherapy on depression and anxiety levels among 46 postmenopausal women who were administered 2% inhaled lavender essence or distilled water 20 min in the evening before bedtime for 4 consecutive weeks.	Depression and anxiety mean scores decreased in the lavender group compared with controls, providing evidence that lavender aromatherapy may be an effective non-invasive treatment during the postmenopausal stage.
**Roseroot**			
Mao J.J. et al. 2014 [249]	A randomized, double-blind, placebo-controlled, parallel-group study clinical trial	Evaluation of the safety and efficacy of *R. rosea* (extract 340–1360 mg daily) versus sertraline or placebo therapy in 57 subjects with major depressive disorder (MDD) in a 12-week treatment.	Although *R. rosea* produced less antidepressant effect versus sertraline, it also resulted in significantly fewer adverse events and was better tolerated. These findings suggest that *R. rosea*, although less effective than sertraline, may have a more favourable risk–benefit ratio for people with mild to moderate depression.
**Ginkgo**			
Liang Z.H. et al. 2019 [250]	A randomized, double-blind clinical trial	Evaluation of the efficacy of ginkgo extract (40 mg three times a day) as augmentation of treatment in 80 venlafaxine-treated (75–225 mg/day) post-stroke depression (PSD) patients. Moreover, serum 5-hydroxytryptamine (5-HT) and BDNF levels were measured before and after treatment.	Patients receiving ginkgo extract plus venlafaxine had significantly lower mean depression scale scores. Meanwhile, compared to the control group, patients in the experimental group additionally receiving ginkgo extract had significantly higher 5-HT levels and BDNF levels and required a lower dose of venlafaxine and experienced fewer adverse events. The results showed that ginkgo extract was a good augmentation of venlafaxine in the treatment of PSD.
Dai C.X. et al. 2018 [251]	A randomized clinical trial	Evaluation of the efficacy of ginkgo extract (EGb; 19.2 mg three times daily) as adjunctive treatment in 136 elderly depressed patients treated with citalopram (Cit; 20 mg daily) and the effect on serum glioma-derived protein (S100B) expression, which is reduced in depressive disorders.	The time to onset of efficacy was significantly shorter in the EGb + Cit group. After treatment, S100B levels decreased, and the change in S100B levels in the EGb + Cit group was greater than in the Cit group. EGb, as an adjunctive treatment, can effectively improve depressive symptoms and reduce serum S100B expression, which is a marker of brain damage, suggesting that EGb restores neurological function when treating depression in elderly patients.
**Korean ginseng**			
Jeong H.G. et al. 2015 [252]	An open-label, prospective clinical study	Evaluation of the efficacy and tolerability of adjuvant treatment with Korean red ginseng (3 g/day for 8 weeks) in 25 female patients with residual symptoms of major depression.	Subjects reported a significant reduction in depressive symptoms over 8 weeks. The results suggest that Korean red ginseng is effective as an adjunctive treatment for patients with residual symptoms of major depression.
Lee K.H. et al. 2020 [253]	An open-label, prospective clinical study	Evaluation of the efficacy and tolerability of Korean red ginseng supplementation (RGA; 2 g/day for 6 weeks) in major depressive disorder (MDD) of 36 difficult-to-treat patients who were currently on antidepressant monotherapy (duloxetine, venlafaxine, paroxetine, sertraline, fluoxetine, escitalopram, mirtazapine).	The study demonstrated the presumed efficacy and tolerability of RGA in the treatment of MDD, which is difficult to treat in clinical practice. Remission rates were significantly better in patients treated with RGA.
**Borage**			
Sayyah M. et al. 2006 [254]	A randomized, double-blind, placebo-controlled clinical trial	The evaluation of the efficacy of an aqueous extract of *E. amoenum* (375 mg daily for 6 weeks) in 35 patients with mild to moderate major depressive disorder.	At week 4, the extract showed a significant advantage over the placebo in reducing depressive symptoms. The effect on anxiety was not significant.
Najafabady M.T. et al. 2019 [255]	A double-blind, randomized clinical trial	The aim of the study was to compare the effects of *Echium amoenum* extract (500 mg daily for 8 weeks) with fluoxetine on depression in 72 menopausal women.	At the start of the study, a comparison of the mean depression scores in the two intervention and control groups showed no significant difference. However, a statistically significant difference was observed in the mean depression score in both groups at week 4 of the intervention. No statistically significant difference was observed between the mean depression scores of the two groups at the end of the study.
Saiiah Bargard M. et al. 2004 [256]	A double-blind, randomized clinical trial	Evaluation of the efficacy and safety of an aqueous extract of *Echium amoenum* (375 mg daily for 6 weeks) in 35 patients with mild to moderate major depressive disorder (MDD).	At week 4 of the study, the extract was superior to the placebo, and at week 6, the difference was marginally insignificant. *Echium amoenum* extract did not cause more side effects than placebo during the study. It was concluded that the aqueous extract of Echium amoenum can be considered an effective and safe treatment for MDD.
**Brahmi**			
Shetty S.K. et al. 2021 [257]	An open-label, prospective clinical study	Evaluation of the efficacy of Brahmi (*Bacopa monnieri*; 500 mg twice daily for 30 days) in reducing depression, anxiety and stress among 198 in COVID-19 negative Indian patients aged 12 to 60 years.	The study showed a significant decrease in the severity of depression, anxiety and stress after Brahmi supplementation for 1 month.
Calabrese C. et al. 2008 [258]	A randomized, double-blind, placebo-controlled trial	Evaluation of the effects of *Bacopa monnieri* standardized dry extract (300 mg daily for 12 weeks) on cognitive function and affect, and its safety and tolerability in 54 healthy elderly participants (65 years or older).	Epidemiologic Studies Depression scale (CESD-10) depression scores, joint state plus trait anxiety scores, and decreased heart rate over time for the *Bacopa* group.
**Mimosa tree**			
Xueli S. 2013 [259]	A randomized, parallel controlled clinical trial	Evaluation of the effects of *Albizia julibrissin* flower decoctum (AJF; 30 g, 1 dose/day, decocted in water up to 200 mL, in the afternoon; treatment for 6 weeks) in patients with depression compared to those treated with venlafaxine.	In the treatment group, 8 cases were cured, 20 cases were markedly effective, 10 cases were effective, 12 cases were invalid, the total efficacy was 76.00%. In the control group, 10 cases were cured, 20 cases were markedly effective, 10 cases were effective, 10 cases were invalid, the total efficacy was 80.00%.
**Magnolia bark**			
Xue L. et al. 2020 [260]	A randomized controlled clinical trial	The aim of the study was to investigate whether magnolia tea drunk for 3 weeks (one cup per day, prepared by infusing the tea in 300 mL of hot water for 12 min) has a palliative effect on postnatal depression in 143 postpartum women.	Compared to the control group, the intervention group showed a significant difference in sleep deprivation related to physical symptoms at week 3 post-test. The comparison results also showed alleviation of depressive symptoms at week 3 post-test and week 6 post-test. The results suggested that drinking a single-ingredient magnolia tea for a 3-week duration had positive effects on postpartum women.

## Data Availability

Data sharing not applicable.

## Abbreviations

5-HT	5-hydroxytryptamine (serotonin)
ADRs	adverse drug reactions
AMPA	α-amino-3-hydroxy-5-methyl-4-isoxazolepropionic acid (glutamate receptors)
BDNF	brain derived neurotrophic factor
CNS	central nervous system
COX-2	cyclooxygenase 2
GABA	gamma aminobutyric acid
HPA	hypothalamic-pituitary-axis
SSRIs	selective serotonin reuptake inhibitors
Il	interleukin
INF-γ	interferon-γ
iNOS	inducible nitric oxide synthase
MAH	marketing authorization holder
MAOIs	monoamine oxidase inhibitors
MDD	major depressive disorder
NDRIs	noradrenaline and dopamine reuptake inhibitors
NE	noradrenaline (norepinephrine)
NF-κB	nuclear factor-kappa factor
NMDA	N-methyl-D-aspartate (glutamate receptors)
NRIs	noradrenaline reuptake inhibitors
OTC	over the counter (drugs)
OS	oxidative stress
SNRIs	selective noradrenaline reuptake inhibitors
SJW	St. John’s wort
TCAs	tricyclic antidepressants
TNF-α	tumour necrosis alpha
WHO	World Health Organization

## References

[1] Clack S., Ward T. (2019). The classification and explanation of depression. Behav. Chang..

[2] World Health Organisation ICD-11 for Mortality and Morbidity Statistics. Version: 02/2022. https://icd.who.int/browse11/l-m/en.

[3] Pardhe H.A., Nagalakshmi N.C., Hariprasad M.G., Chourasia P.K., Nandini S. (2020). A review: Medicinal plants with antidepressant properties. Indian J. Neurosci..

[4] Martins J., Brijesh S. (2018). Phytochemistry and pharmacology of anti-depressant medicinal plants: A review. Biomed. Pharmacother..

[5] World Health Organisation Depression. https://www.who.int/news-room/fact-sheets/detail/depression.

[6] Torre J., Vilagut G., Ronaldson A., Serrano-Blanco A., Martín V., Peters M., Valderas J., Dregan A., Alonso J. (2021). Prevalence and variability of current depressive disorder in 27 European countries: A population-based study. Lancet Public Health.

[7] Gałecki P., Samochowiec J., Mikułowska M., Szulc A. (2022). Treatment-Resistant Depression in Poland—Epidemiology and Treatment. J. Clin. Med..

[8] Centers for Disease Control and Prevention National Center for Health Statistics. Anxiety and Depression. https://www.cdc.gov/nchs/covid19/pulse/mental-health.htm.

[9] Chiu E. (2004). Epidemiology of depression in the Asia Pacific region. Australas Psychiatry.

[10] Ogbo F.A., Mathsyaraja S., Koti R.K., Perz J., Page A. (2018). The burden of depressive disorders in South Asia, 1990–2016: Findings from the global burden of disease study. BMC Psychiatry.

[11] Pan American Health Organization and World Health Organization World Mental Health Day: Depression, the Most Common Mental Disorder. https://www3.paho.org/hq/index.php?option=com_content&view=article&id=7305:2012-dia-mundial-salud-mental-depresion-trastorno-mental-mas-frecuente&Itemid=0&lang=en#gsc.tab=0.

[12] Cuadros D.F., Tomita A., Vandormael A., Slotow R., Burns J.K., Tanser F. (2019). Spatial structure of depression in South Africa: A longitudinal panel survey of a nationally representative sample of households. Sci. Rep..

[13] Bedaso A., Mekonnen N., Duko B. (2022). Estimate of the prevalence of depression among older people in Africa: A systematic review and meta-analysis. Aging Ment. Health.

[14] Australian Institute of Health and Welfare Prevalence and Impact of Mental Illness. https://www.aihw.gov.au/mental-health/overview/mental-illness#mental-disorder.

[15] Sarris J., Panossian A., Schweitzer I., Stough C., Scholey A. (2011). Herbal medicine for depression, anxiety and insomnia: A review of psychopharmacology and clinical evidence. Eur. Neuropsychopharmacol..

[16] Bernaras E., Jaureguizar J., Garaigordobil M. (2019). Child and Adolescent Depression: A Review of theories, evaluation instruments, prevention programs, and treatments. Front. Psychol..

[17] Deverteuil R.L., Lehmann H.E. (1958). Therapeutic trial of iproniazid (marsilid) in depressed and apathetic patients. Can. Med. Assoc. J..

[18] Kuhn R. (1958). The treatment of depressive states with G 22355 (imipramine hydrochloride). Am. J. Psychiatry.

[19] Schildkraut J.J., Kety S.S. (1967). Biogenic amines and emotion. Science.

[20] Qin D.D., Rizak J., Feng X.L., Yang S.C., Lü L.B., Pan L., Yin Y., Hu X.T. (2016). Prolonged secretion of cortisol as a possible mechanism underlying stress and depressive behaviour. Sci. Rep..

[21] Herbert J. (2013). Cortisol and depression: Three questions for psychiatry. Psychol. Med..

[22] Mikulska J., Juszczyk G., Gawrońska-Grzywacz M., Herbet M. (2021). HPA Axis in the pathomechanism of depression and schizophrenia: New therapeutic strategies based on its participation. Brain Sci..

[23] Jesulola E., Micalos P., Baguley I.J. (2018). Understanding the Pathophysiology of Depression: From Monoamines to the Neurogenesis Hypothesis Model—Are We There Yet?. Behav. Brain Res..

[24] Lee C.H., Giuliani F. (2019). The Role of Inflammation in Depression and Fatigue. Front. Immunol..

[25] Wohleb E.S., Franklin T., Iwata M., Duman R.S. (2016). Integrating Neuroimmune Systems in the Neurobiology of Depression. Nat. Rev. Neurosci..

[26] Beurel E., Toups M., Nemeroff C.B. (2020). The bidirectional relationship of depression and inflammation: Double trouble. Neuron.

[27] Więdłocha M., Marcinowicz P., Krupa R., Janoska-Jaździk M., Janus M., Dębowska W., Mosiołek A., Waszkiewicz N., Szulc A. (2018). Effect of antidepressant treatment on peripheral inflammation markers—A meta-analysis. Prog. Neuropsychopharmacol. Biol. Psychiatry.

[28] Savitz J. (2017). Role of kynurenine metabolism pathway activation in major depressive disorders. Curr. Top. Behav. Neurosci..

[29] Berk M., Williams L.J., Jacka F.N., O’Neil A., Pasco J.A., Moylan S., Allen N.B., Stuart A.L., Hayley A.C., Byrne M.L. (2013). So depression is an inflammatory disease, but where does the inflammation come from?. BMC Med..

[30] Salim S. (2017). Oxidative stress and the central nervous system. J. Pharmacol. Exp. Ther..

[31] Jimenez-Fernandez S., Gurpegui M., Diaz-Atienza F., Perez-Costillas L., Gerstenberg M., Correll C.U. (2015). Oxidative stress and antioxidant parameters in patients with major depressive disorder compared to healthy controls before and after antidepressant treatment: Results from a meta-analysis. J. Clin. Psychiatry.

[32] Guilliams T.G., Edwards L. (2010). Chronic stress and the HPA axis: Clinical assessment and therapeutic considerations. Standard.

[33] Carabotti M., Scirocco A., Maselli M.A., Severi C. (2015). The Gut-Brain Axis: Interactions between enteric microbiota, central and enteric nervous systems. Ann. Gastroenterol..

[34] Cryan J.F., O’Riordan K.J., Cowan C.S.M., Sandhu K.V., Bastiaanssen T.F.S., Boehme M., Codagnone M.G., Cussotto S., Fulling C., Golubeva A.V. (2019). The Microbiota-Gut-Brain Axis. Physiol. Rev..

[35] Liang S., Wu X., Hu X., Wang T., Jin F. (2018). Recognizing depression from the microbiota–gut–brain axis. Int. J. Mol. Sci..

[36] Alvarez-Mon M.A., Gómez A.M., Orozco A., Lahera G., Sosa M.D., Diaz D., Auba E., Albillos A., Monserrat J., Alvarez-Mon M. (2019). Abnormal distribution and function of circulating monocytes and enhanced bacterial translocation in major depressive disorder. Front. Psychiatry.

[37] Strandwitz P. (2018). Neurotransmitter modulation by the gut microbiota. Brain Res..

[38] Bromberger J.T., Schott L.L., Kravitz H.M., Sowers M., Avis N.E., Gold E.B., Randolph J.F., Matthews K.A. (2010). Longitudinal change in reproductive hormones and depressive symptoms across the menopausal transition: Results from the Study of Women’s Health Across the Nation (SWAN). Arch. Gen. Psychiatry.

[39] Soares C.N., Zitek B. (2008). Reproductive hormone sensitivity and risk for depression across the female life cycle: A continuum of vulnerability?. J. Psychiatry Neurosci..

[40] Sullivan P.F., Neale M.C., Kendler K.S. (2000). Genetic epidemiology of major depression: Review and meta-analysis. Am. J. Psychiatry.

[41] Menke A., Klengel T., Binder E.B. (2012). Epigenetics, depression and antidepressant treatment. Curr. Pharm. Des..

[42] Krishnan V., Nestler E.J. (2008). The molecular neurobiology of depression. Nature.

[43] Monteggia L.M., Luikart B., Barrot M., Theobold D., Malkovska I., Nef S., Parada L.F., Nestler E.J. (2007). Brain-derived neurotrophic factor conditional knockouts show gender differences in depression-related behaviors. Biol. Psychiatry.

[44] Eisch A.J., Bolaños C.A., de Wit J., Simonak R.D., Pudiak C.M., Barrot M., Verhaagen J., Nestler E.J. (2003). Brain-derived neurotrophic factor in the ventral midbrain-nucleus accumbens pathway: A role in depression. Biol. Psychiatry..

[45] Duman R.S., Voleti B. (2012). Signaling pathways underlying the pathophysiology and treatment of depression: Novel mechanisms for rapid-acting agents. Trends Neurosci..

[46] Shadrina M., Bondarenko E.A., Slominsky P.A. (2018). Genetics factors in major depression disease. Front. Psychiatry.

[47] Lohoff F.W. (2010). Overview of the genetics of major depressive disorder. Curr. Psychiatry Rep..

[48] Mariani N., Cattane N., Pariante C., Cattaneo A. (2021). Gene expression studies in depression development and treatment: An overview of the underlying molecular mechanisms and biological processes to identify biomarkers. Transl. Psychiatry.

[49] Dupont C., Armant D.R., Brenner C.A. (2009). Epigenetics: Definition, mechanisms and clinical perspective. Semin. Reprod. Med..

[50] Al Aboud N.M., Tupper C., Jialal I. (2022). Genetics, Epigenetic Mechanism. StatPearls.

[51] Penner-Goeke S., Binder E.B. (2019). Epigenetics and depression. Dialogues Clin. Neurosci..

[52] Czarny P., Białek K., Ziółkowska S., Strycharz J., Barszczewska G., Sliwinski T. (2021). The importance of epigenetics in diagnostics and treatment of major depressive disorder. J. Pers. Med..

[53] Blatt S.J. (2004). Experiences of Depression: Theoretical, Clinical, and Research Perspectives.

[54] Tonon A.C., Pilz L.K., Markus R.P., Hidalgo M.P., Elisabetsky. E. (2021). Melatonin and depression: A translational perspective from animal models to clinical studies. Front. Psychiatry.

[55] Wang Y.Q., Jiang Y.J., Zou M.S., Liu J., Zhao H.Q., Wang Y.H. (2022). Antidepressant actions of melatonin and melatonin receptor agonist: Focus on pathophysiology and treatment. Behav. Brain Res..

[56] González-Díaz S.N., Arias-Cruz A., Elizondo-Villarreal B., Monge-Ortega O.P. (2017). Psychoneuroimmunoendocrinology: Clinical implications. World Allergy Organ. J..

[57] Singh T., Williams K. (2006). Atypical depression. Psychiatry.

[58] Munro M., Milne R. (2020). Symptoms and causes of depression, and its diagnosis and management. Nurs. Times.

[59] Kennedy S.H. (2008). Core symptoms of major depressive disorder: Relevance to diagnosis and treatment. Dialogues Clin. Neurosci..

[60] Bhowmik D., Kumar K.P.S., Srivastava S., Paswan S., Dutta A.S. (2012). Depression-symptoms, causes, medications and therapies. Pharma Innov..

[61] Kuria M.W., Ndetei D.M., Obot I.S., Khasakhala L.I., Bagaka B.M., Mbugua M.N., Kamau J. (2012). The association between alcohol dependence and depression before and after treatment for alcohol dependence. ISRN Psychiatry.

[62] Timonen M., Liukkonen T. (2008). Management of depression in adults. BMJ.

[63] Bleakley S. (2009). Review of choice and use of antidepressants. Prog. Neurol. Psychiatry.

[64] Faquih A.E., Memon R.I., Hafeez H., Zeshan M., Naveed S. (2019). A review of novel antidepressants: A guide for clinicians. Cureus.

[65] Cipriani A., Furukawa T.A., Salanti G., Chaimani A., Atkinson L.Z., Ogawa Y., Leucht S., Ruhe H.G., Turner E.H., Higgins J.P.T. (2018). Comparative efficacy and acceptability of 21 antidepressant drugs for the acute treatment of adults with major depressive disorder: A systematic review and network meta-analysis. Lancet.

[66] Ramic E., Prasko S., Gavran L., Spahic E. (2020). Assessment of the antidepressant side effects occurrence in patients treated in primary care. Mater. Sociomed..

[67] Edinoff A.N., Akuly H.A., Hanna T.A., Ochoa C.O., Patti S.J., Ghaffar Y.A., Kaye A.D., Viswanath O., Urits I., Boyer A.G. (2021). Selective Serotonin Reuptake Inhibitors and Adverse Effects: A Narrative Review. Neurol. Int..

[68] Moraczewski J., Aedma K.K. (2022). Tricyclic Antidepressants. StatPearls.

[69] Gautam S., Jain A., Gautam M., Vahia V.N., Grover S. (2017). Clinical practice guidelines for the management of depression. Indian J. Psychiatry.

[70] Practice Guideline for the Treatment of Patients with Major Depressive Disorder. https://psychiatryonline.org/pb/assets/raw/sitewide/practice_guidelines/guidelines/mdd.pdf.

[71] Almeida S.S., Zizzi F.B., Cattaneo A., Comandini A., Di Dato G., Lubrano E., Pellicano C., Spallone V., Tongiani S., Torta R. (2020). Management and treatment of patients with major depressive disorder and chronic diseases: A multidisciplinary approach. Front. Psychol..

[72] American Psychological Association (2019). Clinical Practice Guideline for the Treatment of Depression across Three Age Cohorts. https://www.apa.org/depression-guideline.

[73] Samochowiec J., Dudek D., Mazur J.K., Murawiec S., Rymaszewska J., Cubała W.J., Heitzman J., Szulc A., Bała M., Gałecki P. (2021). Pharmacological treatment of a depressive episode and recurrent depressive disorder—Guidelines of the Polish Psychiatric Association and the National Consultant for Adult Psychiatry. Psychiatry Pol..

[74] Farah W.H., Alsawas M., Mainou M., Alahdab F., Farah M.H., Ahmed A.T., Mohamed E.A., Almasri J., Gionfriddo M.R., Castaneda-Guarderas A. (2016). Non-pharmacological treatment of depression: A systematic review and evidence map. Evid. Based Med..

[75] Gautam M., Tripathi A., Deshmukh D., Gaur M. (2020). Cognitive behavioral therapy for depression. Indian J. Psychiatry.

[76] Thurfah J.N., Christine, Bagaskhara P.P., Alfian S.D., Puspitasari I.M. (2022). Dietary supplementations and depression. J. Multidiscip. Healthc..

[77] Ortega M.A., Fraile-Martínez Ó., García-Montero C., Alvarez-Mon M.A., Lahera G., Monserrat J., Llavero-Valero M., Gutiérrez-Rojas L., Molina R., Rodríguez-Jimenez R. (2022). Biological role of nutrients, food and dietary patterns in the prevention and clinical management of major depressive disorder. Nutrients.

[78] Rizvi S., Khan A.M. (2019). Use of transcranial magnetic stimulation for depression. Cureus.

[79] Porter R., Linsley K., Ferrier N. (2001). Treatment of severe depression—Non-pharmacological aspects. Adv. Psychiatr. Treat..

[80] Chen J.J., Zhao L.B., Liu Y.Y., Fan S.H., Xie P. (2017). Comparative efficacy and acceptability of electroconvulsive therapy versus repetitive transcranial magnetic stimulation for major depression: A systematic review and multiple-treatments meta-analysis. Behav. Brain Res..

[81] Micallef-Trigona B. (2014). Comparing the effects of repetitive transcranial magnetic stimulation and electroconvulsive therapy in the treatment of depression: A systematic review and meta-analysis. Depress Res. Treat..

[82] Iacobucci G. (2019). NHS prescribed record number of antidepressants last year. BMJ.

[83] Pratt L.A., Brody D.J., Gu Q. (2017). Antidepressant use among persons aged 12 and over: United States, 2011–2014. CHS Data Brief.

[84] Reinert M., Fritze D., Nguyen T. The State of Mental Health in America 2022. https://mhanational.org/sites/default/files/2022%20State%20of%20Mental%20Health%20in%20America.pdf.

[85] Lewer D., O’Reilly C., Mojtabai R., Evans-Lacko S. (2015). Antidepressant use in 27 European countries: Associations with sociodemographic, cultural and economic factors. Br. J. Psychiatry.

[86] Sisay T., Wami R. (2021). Adverse drug reactions among major depressive disorders: Patterns by age and gender. Heliyon.

[87] Degner D., Grohmann R., Kropp S., Rüther E., Bender S., Engel R.R., Schmidt L.G. (2004). Severe adverse drug reactions of antidepressants: Results of the German multicenter drug surveillance program AMSP. Pharmacopsychiatry.

[88] Uher R., Farmer A., Henigsberg N., Rietschel M., Mors O., Maier W., Kozel D., Hauser J., Souery D., Placentino A. (2009). Adverse reactions to antidepressants. Br. J. Psychiatry.

[89] Jiao Y., Wickett N.J., Ayyampalayam S., Chanderbali A.S., Landherr L., Ralph P.E., Tomsho L.P., Hu Y., Liang H., Soltis P.S. (2011). Ancestral polyploidy in seed plants and angiosperms. Nature.

[90] Sahoo S. (2018). A review of some medicinal plants used for nervous system. J. Med. Plants Stud..

[91] Ulrich-Merzenich G., Panek D., Zeitler H., Vetter H., Wagner H. (2010). Drug development from natural products: Exploiting synergistic effects. Indian J. Exp. Biol..

[92] Wagner H., Ulrich-Merzenich G. (2009). Synergy research: Approaching a new generation of phytopharmaceuticals. Phytomedicine.

[93] Wink M. (2015). Modes of action of herbal medicines and plant secondary metabolites. Medicines.

[94] Kessler R.C., Soukup J., Davis R.B., Foster D.F., Wilkey S.A., Van Rompay M.I., Eisenberg D.M. (2001). The use of complementary and alternative therapies to treat anxiety and depression in the United States. Am. J. Psychiatry.

[95] Elkins G., Rajab M.H., Marcus J. (2005). Complementary and alternative medicine use by psychiatric inpatients. Psychol. Rep..

[96] Kumar V. (2006). Potential medicinal plants for CNS disorders: An overview. Phytother Res.

[97] Peschel W. (2014). The use of community herbal monographs to facilitate registrations and authorisations of herbal medicinal products in the European Union 2004–2012. J. Ethnopharmacol..

[98] Moreira D.L., Teixeira S.S., Monteiro M.H.D., de-Oliveira A.C.A.X., Paumgartten F.J.R. (2014). Traditional use and safety of herbal medicines. Rev. Bras. Farmacogn..

[99] Papakostas G.I. (2010). The efficacy, tolerability, and safety of contemporary antidepressants. J. Clin. Psychiatry.

[100] Baldwin D.S., Montgomery S.A., Nil R., Lader M. (2007). Discontinuation symptoms in depression and anxiety disorders. Int. J. Neuropsychopharmacol..

[101] Wilson E., Lader M. (2015). A review of the management of antidepressant discontinuation symptoms. Ther. Adv. Psychopharmacol..

[102] Alvarez-Mon M.A., Ortega M.A., García-Montero C., Fraile-Martinez O., Monserrat J., Lahera G., Mora F., Rodriguez-Quiroga A., Fernandez-Rojo S., Quintero J. (2021). Exploring the role of nutraceuticals in major depressive disorder (MDD): Rationale, state of the art and future prospects. Pharmaceuticals.

[103] Wang Y., Su C., Zhang B., Niu Y., Ren R., Zhao X., Yang L., Zhang W., Ma X. (2021). Biological activity, hepatotoxicity, and structure-activity relationship of kavalactones and flavokavins, the two main bioactive components in kava (*Piper methysticum*). Evid. Based Complement. Altern. Med..

[104] Staines S.S. (2011). Herbal medicines: Adverse effects and drug-herbs interactions. J. Malta Coll. Pharm. Pract..

[105] Henderson L., Yue Q.Y., Bergquist C., Gerden B., Arlett P. (2002). St. John’s wort (*Hypericum perforatum*): Drug interactions and clinical outcomes. Br. J. Clin. Pharmacol..

[106] Canenguez Benitez J.S., Hernandez T.E., Sundararajan R., Sarwar S., Arriaga A.J., Khan A.T., Matayoshi A., Quintanilla H.A., Kochhar H., Alam M. (2022). Advantages and disadvantages of using St. John’s Wort as a treatment for depression. Cureus.

[107] Ekor M. (2014). The growing use of herbal medicines: Issues relating to adverse reactions and challenges in monitoring safety. Front. Pharmacol..

[108] Hammerness P., Basch E., Ulbricht C., Barrette P., Foppa I., Basch S., Bent S., Boon H., Ernst E., Natural Standard Research Collaboration (2003). St John’s wort: A systematic review of adverse effects and drug interactions for the consultation psychiatrist. Psychosomatics.

[109] Joshi K.G., Faubion M.D. (2005). Mania and psychosis associated with St. John’s wort and ginseng. Psychiatry.

[110] Nicolussi S., Drewe J., Butterweck V., Zu Schwabedissen H.E.M. (2020). Clinical relevance of St. John’s wort drug interactions revisited. Br. J. Pharmacol..

[111] Grimstein M., Huang S.M. (2018). A regulatory science viewpoint on botanical-drug interactions. J. Food Drug Anal..

[112] Moore L.B., Goodwin B., Jones S.A., Wisely G.B., Serabjit-Singh C.J., Willson T.M., Collins J.L., Kliewer S.A. (2000). St. John’s wort induces hepatic drug metabolism through activation of the pregnane X receptor. Proc. Natl. Acad. Sci. USA.

[113] Markowitz J.S., Donovan J.L., DeVane C.L., Taylor R.M., Ruan Y., Wang J.S., Chavin K.D. (2003). Effect of St John’s wort on drug metabolism by induction of cytochrome P450 3A4 enzyme. JAMA.

[114] Kleemann B., Loos B., Scriba T.J., Lang D., Davids L.M. (2014). St. John’s wort (*Hypericum perforatum* L.) photomedicine: Hypericin-photodynamic therapy induces metastatic melanoma cell death. PLoS ONE.

[115] Vollmer J.J., Rosenson J. (2004). Chemistry of St. John’s wort: Hypericin and hyperforin. J. Chem. Educ..

[116] Moshiri M., Vahabzadeh M., Hosseinzadeh H. (2015). Clinical applications of saffron (*Crocus sativus*) and its constituents: A review. Drug Res..

[117] Barnes J. (2022). Saffron. J. Prim. Health Care.

[118] Zam W., Quispe C., Sharifi-Rad J., López M.D., Schoebitz M., Martorell M., Sharopov F., Fokou P.V.T., Mishra A.P., Chandran D. (2022). An updated review on the properties of *Melissa officinalis* L.: Not exclusively anti-anxiety. Front. Biosci..

[119] Block K.I., Gyllenhaal C., Mead M.N. (2004). Safety and efficacy of herbal sedatives in cancer care. Integr. Cancer Ther..

[120] Unger M. (2013). Pharmacokinetic drug interactions involving *Ginkgo biloba*. Drug Metab. Rev..

[121] Nguyen T., Alzahrani T. (2022). Ginkgo Biloba. StatPearls.

[122] Sadler C., Charrois T.L., Vohra S. (2006). *Gingko biloba*: Practical management of adverse effects and drug interactions. Canadian Pharm. J. Rev. Pharm. Can..

[123] Kiefer D., Pantuso T. (2003). Panax ginseng. Am. Fam. Phys..

[124] Tao H., Wu X., Cao J., Peng Y., Wang A., Pei J., Xiao J., Wang S., Wang Y. (2019). Rhodiola species: A comprehensive review of traditional use, phytochemistry, pharmacology, toxicity, and clinical study. Med. Res. Rev..

[125] Sarrica A., Kirika N., Romeo M., Salmona M., Diomede L. (2018). Safety and toxicology of magnolol and honokiol. Planta Med..

[126] Sayyah M., Siahpoosh A., Khalili H., Malayeri A., Samaee H. (2012). A double-blind, placebo-controlled study of the aqueous extract of *Echium amoenum* for patients with general anxiety disorder. Iran J. Pharm. Res..

[127] Peth-Nui T., Wattanathorn J., Muchimapura S., Tong-Un T., Piyavhatkul N., Rangseekajee P., Ingkaninan K., Vittaya-Areekul S. (2012). Effects of 12-week *Bacopa monnieri* consumption on attention, cognitive processing, working memory, and functions of both cholinergic and monoaminergic systems in healthy elderly volunteers. Evid. Based Complement. Alternat. Med..

[128] Sanaye M.M., Joglekar C.S., Pagare N.P. (2015). Mimosa—A brief overview. J. Pharmacogn. Phytochem..

[129] Wang Y.S., Shen C.Y., Jiang J.G. (2019). Antidepressant active ingredients from herbs and nutraceuticals used in TCM: Pharmacological mechanisms and prospects for drug discovery. Pharmacol. Res..

[130] Farahani M.S., Bahramsoltani R., Farzaei M.H., Abdollahi M., Rahimi R. (2015). Plant-derived natural medicines for the management of depression: An overview of mechanisms of action. Rev. Neurosci..

[131] Zhang Z., Deng T., Wu M., Zhu A., Zhu G. (2019). Botanicals as modulators of depression and mechanisms involved. Chin. Med..

[132] Lundstrom K., Pham H.T., Dinh L.D. (2017). Interaction of plant extracts with central nervous system receptors. Medicines.

[133] Parveen A., Parveen B., Parveen R., Ahmad S. (2015). Challenges and guidelines for clinical trial of herbal drugs. J. Pharm. Bioallied Sci..

[134] Jachak S.M., Saklani A. (2007). Challenges and opportunities in drug discovery from plants. Curr. Sci..

[135] Williams R., Münch G., Gyengesi E., Bennett L. (2014). *Bacopa monnieri* (L.) exerts anti-inflammatory effects on cells of the innate immune system in vitro. Food Funct..

[136] Nemetchek M.D., Stierle A.A., Stierle D.B., Lurie D.I. (2017). The Ayurvedic plant *Bacopa monnieri* inhibits inflammatory pathways in the brain. J. Ethnopharmacol..

[137] Mathur A.S., Verma S.K., Purohit R., Singh S.K., Mathur D., Prasad G.K., Dua V.K. (2010). Pharmacological investigation of *Bacopa monnieri* on the basis of antioxidant, antimicrobial and anti-inflammatory properties. J. Chem. Pharm. Res..

[138] Rai K., Gupta N.S., Dharamdasani L., Nair P., Bodhankar P. (2017). *Bacopa monnieri*: A wonder drug changing fortune of people. IJASBT.

[139] Bhattacharya S.K., Bhattacharya A., Kumar A., Ghosal S. (2000). Antioxidant activity of *Bacopa monniera* in rat frontal cortex, striatum and hippocampus. Phytother. Res..

[140] Jin J., Boersch M., Nagarajan A., Davey A.K., Zunk M. (2020). Antioxidant properties and reported ethnomedicinal use of the genus *Echium* (Boraginaceae). Antioxidants.

[141] Naseri N., Kalantar K., Amirghofran Z. (2018). Anti-inflammatory activity of *Echium amoenum* extract on macrophages mediated by inhibition of inflammatory mediators and cytokines expression. Res. Pharm. Sci..

[142] Das Noor-E-Tabassum R., Lami M.S., Chakraborty A.J., Mitra S., Tallei T.E., Idroes R., Mohamed A.A., Hossain M.J., Dhama K., Mostafa-Hedeab G. (2022). *Ginkgo biloba*: A treasure of functional phytochemicals with multimedicinal applications. Evid. Based Complement. Alternat. Med..

[143] Kaur S., Sharma N., Nehru B. (2018). Anti-inflammatory effects of *Ginkgo biloba* extract against trimethyltin-induced hippocampal neuronal injury. Inflammopharmacology.

[144] Gargouri B., Carstensen J., Bhatia H.S., Huell M., Dietz G.P.H., Fiebich B.L. (2018). Anti-neuroinflammatory effects of *Ginkgo biloba* extract EGb761 in LPS-activated primary microglial cells. Phytomedicine.

[145] Li M., Li B., Hou Y., Tian Y., Chen L., Liu S., Zhang N., Dong J. (2019). Anti-inflammatory effects of chemical components from *Ginkgo biloba* L. male flowers on lipopolysaccharide-stimulated RAW264.7 macrophages. Phytother. Res..

[146] Piazza S., Pacchetti B., Fumagalli M., Bonacina F., Dell’Agli M., Sangiovanni E. (2019). Comparison of two *Ginkgo biloba* L. extracts on oxidative stress and inflammation markers in human endothelial cells. Mediat. Inflamm..

[147] Barbalho S.M., Direito R., Laurindo L.F., Marton L.T., Guiguer E.L., Goulart R.A., Tofano R.J., Carvalho A.C.A., Flato U.A.P., Tofano V.A.C. (2022). *Ginkgo biloba* in the aging process: A narrative review. Antioxidants.

[148] An M.Y., Lee S.R., Hwang H.J., Yoon J.G., Lee H.J., Cho J.A. (2021). Antioxidant and anti-inflammatory effects of korean black ginseng extract through ER stress pathway. Antioxidants.

[149] Park J., Cho J.Y. (2009). Anti-inflammatory effects of ginsenosides from Panax ginseng and their structural analogs. Afr. J. Biotechnol..

[150] Pandur E., Balatinácz A., Micalizzi G., Mondello L., Horváth A., Sipos K., Horváth G. (2021). Anti-inflammatory effect of lavender (*Lavandula angustifolia* Mill.) essential oil prepared during different plant phenophases on THP-1 macrophages. BMC Complement. Med. Ther..

[151] Silva G.L., Luft C., Lunardelli A., Amaral R.H., Melo D.A., Donadio M.V., Nunes F.B., de Azambuja M.S., Santana J.C., Moraes C.M. (2015). Antioxidant, analgesic and anti-inflammatory effects of lavender essential oil. An. Acad. Bras. Cienc..

[152] Cardia G.F., Silva-Filho S.E., Silva E.L., Uchida N.S., Cavalcante H.A., Cassarotti L.L., Salvadego V.E., Spironello R.A., Bersani-Amado C.A., Cuman R.K. (2018). Effect of lavender (*Lavandula angustifolia*) essential oil on acute inflammatory response. Evid. Based Complement. Alternat. Med..

[153] Świąder K., Startek K., Wijaya C.H. (2019). The therapeutic properties of lemon balm (*Melissa officinalis* L.): Reviewing novel findings and medical indications. J. Appl. Bot. Food Qual..

[154] Draginic N., Andjic M., Jeremic J., Zivkovic V., Kocovic A., Tomovic M., Bozin B., Kladar N., Bolevich S., Jakovljevic V. (2022). Anti-inflammatory and antioxidant effects of *Melissa officinalis* extracts: A comparative study. Iran J. Pharm. Res..

[155] Miraj S., Rafieian-Kopaei, Kiani S. (2017). *Melissa officinalis* L: A review study with an antioxidant prospective. J. Evid. Based Complement. Altern. Med..

[156] Eliaz I. (2014). Honokiol research review. A promising extract with multiple applications. Nat. Med. J..

[157] Walker J.M., Maitra A., Walker J., Ehrnhoefer-Ressler M.M., Inui T., Somoza V. (2013). Identification of *Magnolia officinalis* L. bark extract as the most potent anti-inflammatory of four plant extracts. Am. J. Chin. Med..

[158] Lee J., Jung E., Park J., Jung K., Lee S., Hong S., Park J., Park E., Kim J., Park S. (2005). Anti-inflammatory effects of magnolol and honokiol are mediated through inhibition of the downstream pathway of MEKK-1 in NF-kappaB activation signaling. Planta Med..

[159] Ramachandran C., Wilk B., Melnick S.J., Eliaz I. (2017). Synergistic antioxidant and anti-inflammatory effects between modified citrus pectin and honokiol. Evid. Based Complement. Alternat. Med..

[160] Kokila K., Priyadharshini S.D., Sujatha V. (2013). Phytopharmacological properties of Albizia species: A review. Int. J. Pharm. Pharm. Sci..

[161] Balkrishna A., Sakshi, Chauhan M., Dabas A., Arya V. (2022). A comprehensive insight into the phytochemical, pharmacological potential, and traditional medicinal uses of *Albizia lebbeck* (L.) Benth. Evid. Based Complement. Alternat. Med..

[162] Babu N.P., Pandikumar P., Ignacimuthu S. (2009). Anti-inflammatory activity of *Albizia lebbeck* Benth., an ethnomedicinal plant, in acute and chronic animal models of inflammation. J. Ethnopharmacol..

[163] Kuum M.G.M., Guemmogne R.J.T., Ndzana M.T.B., Tchadji J.C., Lissom A., Dimo T. (2018). Anti-inflammatory effects of the stem barks from *Albizia ferruginea* (Mimosaceae) on chronic inflammation induced in rats. Int. J. Innov. Res. Med. Sci..

[164] Meshram G.G., Kumar A., Rizvi W., Tripathi C.D., Khan R.A. (2015). Evaluation of the anti-inflammatory activity of the aqueous and ethanolic extracts of the leaves of *Albizzia lebbeck* in rats. J. Tradit. Complement. Med..

[165] Bakasatae N., Kunworarath N., Takahashi Yupanqui C., Voravuthikunchai S.P., Joycharat N. (2018). Bioactive components, antioxidant, and anti-inflammatory activities of the wood of *Albizia myriophylla*. Rev. Bras. Farmacogn..

[166] Pu W.L., Zhang M.Y., Bai R.Y., Sun L.K., Li W.H., Yu Y.L., Zhang Y., Song L., Wang Z.X., Peng Y.F. (2020). Anti-inflammatory effects of *Rhodiola rosea* L.: A review. Biomed. Pharmacother..

[167] Kosakowska O., Bączek K., Przybył J.L., Pióro-Jabrucka E., Czupa W., Synowiec A., Gniewosz M., Costa R., Mondello L., Węglarz Z. (2018). Antioxidant and antibacterial activity of roseroot (*Rhodiola rosea* L.) dry extracts. Molecules.

[168] Lee Y., Jung J.C., Jang S., Kim J., Ali Z., Khan I.A., Oh S. (2013). Anti-inflammatory and neuroprotective effects of constituents isolated from *Rhodiola rosea*. Evid. Based Complement Alternat. Med..

[169] Zeinali M., Zirak M.R., Rezaee S.A., Karimi G., Hosseinzadeh H. (2019). Immunoregulatory and anti-inflammatory properties of *Crocus sativus* (Saffron) and its main active constituents: A review. Iran J. Basic Med. Sci..

[170] Ebrahimi F., Sahebkar A., Aryaeian N., Pahlavani N., Fallah S., Moradi N., Abbasi D., Hosseini A.F. (2019). Effects of saffron supplementation on inflammation and metabolic responses in type 2 diabetic patients: A randomized, double-blind, placebo-controlled trial. Diabetes Metab. Syndr. Obes..

[171] Ghobadi H., Abdollahi N., Madani H., Aslani M.R. (2022). Effect of crocin from saffron (*Crocus sativus* L.) supplementation on oxidant/antioxidant markers, exercise capacity, and pulmonary function tests in COPD patients: A randomized, double-blind, placebo-controlled trial. Front. Pharmacol..

[172] Bahraini M., Hosseini S.A., Cheraghian B., Shoushtari M.H. (2020). Effect of saffron on anti-inflammatory and oxidative stress in asthma. Int. J. Pharm. Phytopharm. Res..

[173] Poursamimi J., Shariati-Sarabi Z., Tavakkol-Afshari J., Mohajeri S.A., Mohammadi M. (2020). *Crocus Sativus* (Saffron): An immunoregulatory factor in the autoimmune and non-autoimmune diseases. Iran J. Allergy Asthma Immunol..

[174] Olajide O.A. (2009). Inhibitory effects of St. John’s Wort on inflammation: Ignored potential of a popular herb. J. Diet Suppl..

[175] Tedeschi E., Menegazzi M., Margotto D., Suzuki H., Förstermann U., Kleinert H. (2003). Anti-inflammatory actions of St. John’s wort: Inhibition of human inducible nitric-oxide synthase expression by down-regulating signal transducer and activator of transcription-1alpha (STAT-1alpha) activation. J. Pharmacol. Exp. Ther..

[176] Koeberle A., Rossi A., Bauer J., Dehm F., Verotta L., Northoff H., Sautebin L., Werz O. (2011). Hyperforin, an anti-inflammatory constituent from St. John’s Wort, inhibits microsomal prostaglandin E(2) synthase-1 and suppresses prostaglandin E(2) formation in vivo. Front. Pharmacol..

[177] Bonaterra G.A., Schwendler A., Hüther J., Schwarzbach H., Schwarz A., Kolb C., Abdel-Aziz H., Kinscherf R. (2018). Neurotrophic, cytoprotective, and anti-inflammatory effects of St. John’s Wort extract on differentiated mouse hippocampal HT-22 neurons. Front. Pharmacol..

[178] Brady K.T., Verduin M.L. (2005). Pharmacotherapy of comorbid mood, anxiety, and substance use disorders. Subst. Use Misuse.

[179] Butterweck V., Schmidt M. (2007). St. John’s wort: Role of active compounds for its mechanism of action and efficacy. Wien. Med. Wochenschr..

[180] Paulke A., Nöldner M., Schubert-Zsilavecz M., Wurglics M. (2008). St. John’s wort flavonoids and their metabolites show antidepressant activity and accumulate in brain after multiple oral doses. Pharmazie.

[181] Butterweck V. (2003). Mechanism of action of St. John’s wort in depression: What is known?. CNS Drugs.

[182] Di Carlo G., Borrelli F., Ernst E., Izzo A.A. (2001). St. John’s wort: Prozac from the plant kingdom. Trends Pharmacol. Sci..

[183] Kholghi G., Arjmandi-Rad S., Zarrindast M.R., Vaseghi S. (2022). St. John’s wort (*Hypericum perforatum*) and depression: What happens to the neurotransmitter systems?. Naunyn. Schmiedebergs. Arch. Pharmacol..

[184] Avila-Sosa R., Nevárez-Moorillón G.V., Ochoa-Velasco C.E., Navarro-Cruz A.R., Hernández-Carranza P., Cid-Pérez T.S. (2022). Detection of saffron’s main bioactive compounds and their relationship with commercial quality. Foods.

[185] Gohari A.R., Saeidnia S., Mahmoodabadi M.K. (2013). An overview on saffron, phytochemicals, and medicinal properties. Pharmacogn. Rev..

[186] Siddiqui S.A., Ali Redha A., Snoeck E.R., Singh S., Simal-Gandara J., Ibrahim S.A., Jafari S.M. (2022). Anti-depressant properties of crocin molecules in saffron. Molecules.

[187] Shafiee M., Arekhi S., Omranzadeh A., Sahebkar A. (2018). Saffron in the treatment of depression, anxiety and other mental disorders: Current evidence and potential mechanisms of action. J. Affect. Disord..

[188] Maqbool Z., Arshad M.S., Ali A., Aziz A., Khalid W., Afzal M.F., Bangar S.P., Addi M., Hano C., Lorenzo J.M. (2022). Potential role of phytochemical extract from saffron in development of functional foods and protection of brain-related disorders. Oxid. Med. Cell. Longev..

[189] Petrisor G., Motelica L., Craciun L.N., Oprea O.C., Ficai D., Ficai A. (2022). *Melissa officinalis*: Composition, pharmacological effects and derived release systems-a review. Int. J. Mol. Sci..

[190] Sharifi-Rad J., Quispe C., Herrera-Bravo J., Akram M., Abbaass W., Semwal P., Painuli S., Konovalov D.A., Alfred M.A., Kumar N.V.A. (2021). Phytochemical constituents, biological activities, and health-promoting effects of the *Melissa officinalis*. Oxid. Med. Cell. Longev..

[191] Wake G., Court J., Pickering A., Lewis R., Wilkins R., Perry E. (2000). CNS acetylcholine receptor activity in European medicinal plants traditionally used to improve failing memory. J. Ethnopharmacol..

[192] Kenda M., Glavač N.K., Nagy M., Dolenc M.S. (2022). Medicinal plants used for anxiety, depression, or stress treatment: An update. Molecules.

[193] Lin S.H., Chou M.L., Chen W.C., Lai Y.S., Lu K.H., Hao C.W., Sheen L.Y. (2015). A medicinal herb, *Melissa officinalis* L. ameliorates depressive-like behavior of rats in the forced swimming test via regulating the serotonergic neurotransmitter. J. Ethnopharmacol..

[194] Białoń M., Krzyśko-Łupicka T., Nowakowska-Bogdan E., Wieczorek P.P. (2019). Chemical composition of two different lavender essential oils and their effect on facial skin microbiota. Molecules.

[195] Prusinowska R., Śmigielski K.B. (2014). Composition, biological properties and therapeutic effects of lavender. A review. Herba Pol..

[196] López V., Nielsen B., Solas M., Ramírez M.J., Jäger A.K. (2017). Exploring pharmacological mechanisms of lavender (*Lavandula angustifolia*) essential oil on central nervous system targets. Front. Pharmacol..

[197] Shamabadi A., Akhondzadeh S. (2022). Bioactive components for depression: Naringin, caffeine, probiotics, saffron and lavender may exert antidepressant effects through inflammation modulation. J. Iran Med. Counc..

[198] Schuwald A.M., Nöldner M., Wilmes T., Klugbauer N., Leuner K., Müller W.E. (2013). Lavender oil-potent anxiolytic properties via modulating voltage dependent calcium channels. PLoS ONE.

[199] Adamczak A., Buchwald W., Gryszczyńska A. (2016). Biometric features and content of phenolic compounds of roseroot (*Rhodiola rosea* L.). Acta Soc. Bot. Pol..

[200] Chiang H.M., Chen H.C., Wu C.S., Wu P.Y., Wen K.C. (2015). Rhodiola plants: Chemistry and biological activity. J. Food Drug Anal..

[201] Amsterdam J.D., Panossian A.G. (2016). *Rhodiola rosea* L. as a putative botanical antidepressant. Phytomedicine.

[202] Konstantinos F., Heun R. (2020). The effects of *Rhodiola rosea* supplementation on depression, anxiety and mood—A systematic review. Glob. Psychiatry.

[203] Stojcheva E.I., Quintela J.C. (2022). The effectiveness of *Rhodiola rosea* L. preparations in alleviating various aspects of life-stress symptoms and stress-induced conditions-encouraging clinical evidence. Molecules.

[204] Fermino B.L., Milanez M.C., de Freitas G.B.L., da Silva W.C.F.N., Pereira R.P., da Rocha J.B.T., Bonini J.S. (2015). *Ginkgo biloba* L.: Phytochemical components and antioxidant activity. AJPP.

[205] Bai S., Zhang X., Chen Z., Wang W., Hu Q., Liang Z., Shen P., Gui S., Zeng L., Liu Z. (2017). Insight into the metabolic mechanism of diterpene ginkgolides on antidepressant effects for attenuating behavioural deficits compared with venlafaxine. Sci. Rep..

[206] Fehske C.J., Leuner K., Müller W.E. (2009). Ginkgo biloba extract (EGb761) influences monoaminergic neurotransmission via inhibition of NE uptake, but not MAO activity after chronic treatment. Pharmacol. Res..

[207] Sloley B.D., Urichuk L.J., Morley P., Durkin J., Shan J.J., Pang P.K., Coutts R.T. (2000). Identification of kaempferol as a monoamine oxidase inhibitor and potential neuroprotectant in extracts of *Ginkgo biloba* leaves. J. Pharm. Pharmacol..

[208] Lü J.M., Yao Q., Chen C. (2009). Ginseng compounds: An update on their molecular mechanisms and medical applications. Curr. Vasc. Pharmacol..

[209] Ratan Z.A., Haidere M.F., Hong Y.H., Park S.H., Lee J.O., Lee J., Cho J.Y. (2021). Pharmacological potential of ginseng and its major component ginsenosides. J. Ginseng Res..

[210] Jin Y., Cui R., Zhao L., Fan J., Li B. (2019). Mechanisms of Panax ginseng action as an antidepressant. Cell Prolif..

[211] Mu D., Ma Q. (2022). A review of antidepressant effects and mechanisms of three common herbal medicines: *Panax ginseng*, *Bupleurum chinense*, and *Gastrodia elata*. CNS Neurol. Disord. Drug Targets.

[212] Hou W., Wang Y., Zheng P., Cui R. (2020). Effects of Ginseng on neurological disorders. Front. Cell. Neurosci..

[213] Abolhassani M. (2010). Antiviral activity of borage (*Echium amoenum*). Arch. Med. Sci..

[214] Zannou O., Pashazadeh H., Ghellam M., Ibrahim S.A., Koca I. (2021). Extraction of anthocyanins from Borage (*Echium amoenum*) flowers using choline chloride and a glycerol-based, deep eutectic solvent: Optimization, antioxidant activity, and in vitro bioavailability. Molecules.

[215] Nouri M., Farajdokht F., Torbati M., Ranjbar F., Hamedyazdan S., Araj-Khodaei M., Sadigh-Eteghad S. (2019). A close look at *Echium amoenum* processing, neuroactive components, and effects on neuropsychiatric disorders. Galen Med. J..

[216] Abdol N., Setorki M. (2020). Evaluation of antidepressant, antianxiolytic, and antioxidant effects of *Echium amoenum* L. extract on social isolation stress of male mice. Iran Red Crescent Med. J..

[217] Jeyasri R., Muthuramalingam P., Suba V., Ramesh M., Chen J.T. (2020). *Bacopa monnieri* and their bioactive compounds inferred multi-target treatment strategy for neurological diseases: A cheminformatics and system pharmacology approach. Biomolecules.

[218] Mathur D., Goyal K., Koul V., Anand A. (2016). The molecular links of re-emerging therapy: A review of evidence of Brahmi (*Bacopa monniera*). Front. Pharmacol..

[219] Liu X., Liu F., Yue R., Li Y., Zhang J., Wang S., Zhang S., Wang R., Shan L., Zhang W. (2013). The antidepressant-like effect of bacopaside I: Possible involvement of the oxidative stress system and the noradrenergic system. Pharmacol. Biochem. Behav..

[220] Sekhar V.C., Viswanathan G., Baby S. (2019). Insights into the molecular aspects of neuroprotective Bacoside A and Bacopaside I. Curr. Neuropharmacol..

[221] Fatima U., Roy S., Ahmad S., Ali S., Elkady W.M., Khan I., Alsaffar R.M., Adnan M., Islam A., Hassan M.I. (2022). Pharmacological attributes of *Bacopa monnieri* extract: Current updates and clinical manifestation. Front. Nutr..

[222] Asgarirad H., Chabra A., Rahimnejad M., Zaghi Hosseinzadeh A., Davoodi A., Azadbakht M. (2018). Comparison of *Albizia Julibressin* and silver sulfadiazine in healing of second and third degree burns. World J. Plast. Surg..

[223] Mahasweta R., Yadav D.K., Kumar B., Kaur J., Patel A.K., Kumar N. (2016). A review on phytochemical and pharmacological studies of *Albizia julibrissin*: An ornamental plant. World J. Pharm. Res..

[224] Patro G., Kumar Bhattamisra S., Kumar Mohanty B. (2016). Effects of *Mimosa pudica* L. leaves extract on anxiety, depression and memory. Avicenna J. Phytomed..

[225] Udyavar S., Kumar K.S., Rai M., Gopalakrishna H.N., Sowmya C.R. (2020). Evaluation of antidepressant activity of ethanolic extract of *Mimosa pudica* in swiss albino mice. Indian J. Pharm. Pharmacol..

[226] Duarte-Filho L.A.M.S., Amariz I.A., Nishimura R.H.V., Massaranduba A.B.R., Menezes P.M.N., Damasceno T.A., Brys I., Rolim L.A., Silva F.S., Ribeiro L.A.A. (2022). β-carboline-independent antidepressant-like effect of the standardized extract of the barks of *Mimosa tenuiflora* (Willd) Poir. occurs via 5-HT2A/2C receptors in mice. J. Psychopharmacol..

[227] Patočka J., Jakl J., Strunecká A. (2006). Expectations of biologically active compounds of the genus Magnolia in biomedicine. J. Appl. Biomed..

[228] Lee Y.J., Lee Y.M., Lee C.K., Jung J.K., Han S.B., Hong J.T. (2011). Therapeutic applications of compounds in the Magnolia family. Pharmacol. Ther..

[229] Yi L.T., Xu Q., Li Y.C., Yang L., Kong L.D. (2009). Antidepressant-like synergism of extracts from magnolia bark and ginger rhizome alone and in combination in mice. Prog. Neuropsychopharmacol. Biol. Psychiatry.

[230] Zhang B., Wang P.P., Hu K.L., Li L.N., Yu X., Lu Y., Chang H.S. (2019). Antidepressant-like effect and mechanism of action of honokiol on the mouse lipopolysaccharide (LPS) depression model. Molecules.

[231] Fan X.X., Sun W.Y., Li Y., Tang Q., Li L.N., Yu X., Wang S.Y., Fan A.R., Xu X.Q., Chang H.S. (2022). Honokiol improves depression-like behaviors in rats by HIF-1α-VEGF signaling pathway activation. Front. Pharmacol..

[232] Tenny S., Varacallo M. (2022). Evidence Based Medicine. StatPearls.

[233] Szajewska H. (2018). Evidence-Based Medicine and clinical research: Both are needed, neither is perfect. Ann. Nutr. Metab..

[234] Murad M.H., Asi N., Alsawas M., Alahdab F. (2016). New evidence pyramid. Evid. Based Med..

[235] Cui Y.H., Zheng Y. (2016). A meta-analysis on the efficacy and safety of St. John’s wort extract in depression therapy in comparison with selective serotonin reuptake inhibitors in adults. Neuropsychiatr. Dis. Treat..

[236] Apaydin E.A., Maher A.R., Shanman R., Booth M.S., Miles J.N., Sorbero M.E., Hempel S. (2016). A systematic review of St. John’s wort for major depressive disorder. Syst. Rev..

[237] Ng Q.X., Venkatanarayanan N., Ho C.Y. (2017). Clinical use of *Hypericum perforatum* (St. John’s wort) in depression: A meta-analysis. J. Affect Disord..

[238] Hausenblas H.A., Heekin K., Mutchie H.L., Anton S. (2015). A systematic review of randomized controlled trials examining the effectiveness of saffron (*Crocus sativus* L.) on psychological and behavioral outcomes. J. Integr. Med..

[239] Hausenblas H.A., Saha D., Dubyak P.J., Anton S.D. (2013). Saffron (*Crocus sativus* L.) and major depressive disorder: A meta-analysis of randomized clinical trials. J. Integr. Med..

[240] Jackson P.A., Forster J., Khan J., Pouchieu C., Dubreuil S., Gaudout D., Moras B., Pourtau L., Joffre F., Vaysse C. (2021). Effects of saffron extract supplementation on mood, well-being, and response to a psychosocial stressor in healthy adults: A randomized, double-blind, parallel group, clinical trial. Front. Nutr..

[241] Akhondzadeh S., Mostafavi S.A., Keshavarz S.A., Mohammadi M.R., Hosseini S., Eshraghian M.R. (2020). A placebo controlled randomized clinical trial of *Crocus sativus* L. (saffron) on depression and food craving among overweight women with mild to moderate depression. J. Clin. Pharm. Ther..

[242] Ghazizadeh J., Sadigh-Eteghad S., Marx W., Fakhari A., Hamedeyazdan S., Torbati M., Taheri-Tarighi S., Araj-Khodaei M., Mirghafourvand M. (2021). The effects of lemon balm (*Melissa officinalis* L.) on depression and anxiety in clinical trials: A systematic review and meta-analysis. Phytother. Res..

[243] Haybar H., Javid A.Z., Haghighizadeh M.H., Valizadeh E., Mohaghegh S.M., Mohammadzadeh A. (2018). The effects of *Melissa officinalis* supplementation on depression, anxiety, stress, and sleep disorder in patients with chronic stable angina. Clin. Nutr. ESPEN.

[244] Heidari M., Soltanpour A., Naseri M., Kazemnezhad A. (2015). The effect of Lemon Balm (*Melissa officinalis*) on depression in patients after coronary artery bypass graft. Iran J. Cardiovasc. Nurs..

[245] Firoozeei T.S., Feizi A., Rezaeizadeh H., Zargaran A., Roohafza H.R., Karimi M. (2021). The antidepressant effects of lavender (*Lavandula angustifolia* Mill.): A systematic review and meta-analysis of randomized controlled clinical trials. Complement. Ther. Med..

[246] Nategh M., Reza H.M., Abbas E., Reza N., Zahra M., Bahman A. (2022). Lavender aromatherapy on anxiety and depression in patients with Acute Coronary Syndrome: A single-blind randomized clinical trial. Front. Nurs..

[247] Tayebi A., Kasra Dehkordi A., Ebadi A., Sahraei H., Einollahi B. (2015). The Effect of aromatherapy with Lavender essential oil on depression, anxiety and stress in hemodialysis patients: A clinical trial. Evid. Based Care J..

[248] Jokar M., Delam H., Bakhtiari S., Paki S., Askari A., Bazrafshan M.R., Shokrpour N. (2020). The effects of inhalation Lavender aromatherapy on postmenopausal women’s depression and anxiety: A randomized clinical trial. JNP.

[249] Mao J.J., Xie S.X., Zee J., Soeller I., Li Q.S., Rockwell K., Amsterdam J.D. (2015). *Rhodiola rosea* versus sertraline for major depressive disorder: A randomized placebo-controlled trial. Phytomedicine.

[250] Liang Z.H., Jia Y.B., Wang M.L., Li Z.R., Li M., Yun Y.L., Zhu R.X. (2019). Efficacy of *Ginkgo Biloba* extract as augmentation of venlafaxine in treating post-stroke depression. Neuropsychiatr. Dis. Treat..

[251] Dai C.X., Hu C.C., Shang Y.S., Xie J. (2018). Role of *Ginkgo biloba* extract as an adjunctive treatment of elderly patients with depression and on the expression of serum S100B. Medicine.

[252] Jeong H.G., Ko Y.H., Oh S.Y., Han C., Kim T., Joe S.H. (2015). Effect of Korean Red Ginseng as an adjuvant treatment for women with residual symptoms of major depression. Asia Pac. Psychiatry.

[253] Lee K.H., Bahk W.M., Lee S.J., Pae C.U. (2020). Effectiveness and tolerability of Korean Red Ginseng augmentation in major depressive disorder patients with difficult-to-treat in routine practice. Clin. Psychopharmacol. Neurosci..

[254] Sayyah M., Sayyah M., Kamalinejad M. (2006). A preliminary randomized double blind clinical trial on the efficacy of aqueous extract of *Echium amoenum* in the treatment of mild to moderate major depression. Prog. Neuropsychopharmacol. Biol. Psychiatry.

[255] Najafabady M.T., Baghbadoranee P.Y., Moghimipour E., Haghighizadeh M.H., Boostani H. (2019). Comparison the effect of *Echium amoenum* extract with fluoxetine on depression in menopausal women. A double-blind randomized controlled trial. Asian J. Pharm..

[256] Bargard M.S., Assadi S., Amini H., Saiiah M., Akhondzadeh S., Kamalinejad M. (2004). Efficacy of aqueous extract of *Echium amoenum* L. in the treatment of mild to moderate major depressive disorder: A randomized double blind clinical trial. J. Med. Plants.

[257] Shetty S.K., Rao P.N., U S., Raj A., Ks S., Sv S. (2021). The effect of Brahmi (*Bacopa monnieri* (L.) Pennell) on depression, anxiety and stress during Covid-19. Eur. J. Integr. Med..

[258] Calabrese C., Gregory W.L., Leo M., Kraemer D., Bone K., Oken B. (2008). Effects of a standardized *Bacopa monnieri* extract on cognitive performance, anxiety, and depression in the elderly: A randomized, double-blind, placebo-controlled trial. J. Altern. Complement. Med..

[259] Shi X., Jiang C., Zhao X., Zhang Y.Q. (2013). Study on the Effects of *Albizia julibrissin* flower on cognitive function and plasma 5-HT, NE and DA in patients with depression: A randomized parallel controlled multicenter clinical trial. J. Pract. Tradit. Chin. Intern. Med..

[260] Xue L., Zhang J., Shen H., Ai L., Wu R. (2020). A randomized controlled pilot study of the effectiveness of magnolia tea on alleviating depression in postnatal women. Food Sci. Nutr..

[261] Panossian A.G., Efferth T., Shikov A.N., Pozharitskaya O.N., Kuchta K., Mukherjee P.K., Banerjee S., Heinrich M., Wu W., Guo D.A. (2021). Evolution of the adaptogenic concept from traditional use to medical systems: Pharmacology of stress- and aging-related diseases. Med. Res. Rev..

[262] Panossian A., Efferth T. (2022). Network pharmacology of adaptogens in the assessment of their pleiotropic therapeutic activity. Pharmaceuticals.

[263] Jawaid T., Gupta R., Siddiqui Z.A. (2011). A review on herbal plants showing antidepressant activity. IJPSR.

[264] Rabiei Z., Rabiei S. (2017). A review on antidepressant effect of medicinal plants. Bangladesh J. Pharmacol..

[265] Taboada T., Alvarenga N.L., Galeano A.K., Arrúa W.J., Campuzano-Bublitz M.A., Kennedy M.L. (2022). In vivo antidepressant-like effect assessment of two Aloysia species in mice and LCMS chemical characterization of ethanol extract. Molecules.

[266] Wang J., Hu D., Hou J., Li S., Wang W., Li J., Bai J. (2018). Ethyl acetate fraction of *Hemerocallis citrina* Baroni decreases tert-butyl hydroperoxide-induced oxidative stress damage in BRL-3A cells. Oxid. Med. Cell. Longev..

[267] Matraszek-Gawron R., Chwil M., Terlecka P., Skoczylas M.M. (2019). Recent studies on anti-depressant bioactive substances in selected species from the genera Hemerocallis and Gladiolus: A systematic review. Pharmaceuticals.

[268] Odhiambo J.A., Siboe G.M., Lukhoba C.W., Dossaji S.F. (2009). Antifungal activity of crude extracts of *Gladiolus dalenii* van geel (iridaceae). Afr. J. Tradit. Complement. Altern. Med..

[269] Agarwa P., Sharma B., Fatima A., Jain S.K. (2014). An update on Ayurvedic herb *Convolvulus pluricaulis* Choisy. Asian Pac. J. Trop. Biomed..

[270] Sharma R., Singla R.K., Banerjee S., Sinha B., Shen B., Sharma R. (2022). Role of Shankhpushpi (*Convolvulus pluricaulis*) in neurological disorders: An umbrella review covering evidence from ethnopharmacology to clinical studies. Neurosci. Biobehav. Rev..

[271] Moragrega I., Ríos J.L. (2021). Medicinal plants in the treatment of depression: Evidence from preclinical studies. Planta Med..

[272] Moragrega I., Ríos J.L. (2022). Medicinal plants in the treatment of depression. II: Evidence from clinical trials. Planta Med..

